# An integrated methodology for assessing the impact of food matrix and gastrointestinal effects on the biokinetics and cellular toxicity of ingested engineered nanomaterials

**DOI:** 10.1186/s12989-017-0221-5

**Published:** 2017-10-13

**Authors:** Glen M. DeLoid, Yanli Wang, Klara Kapronezai, Laura Rubio Lorente, Roujie Zhang, Georgios Pyrgiotakis, Nagarjun V. Konduru, Maria Ericsson, Jason C. White, Roberto De La Torre-Roche, Hang Xiao, David Julian McClements, Philip Demokritou

**Affiliations:** 1 Center for Nanotechnology and Nanotoxicology, HSPH-NIEHS Nanosafety Research Center, Department of Environmental Health, Harvard School of Public Health, Boston, MA 02115 USA; 2 Department of Food Science, University of Massachusetts Amherst, Amherst, MA 01003 USA; 3 Department of Cell Biology, Harvard Medical School, Boston, MA 02115 USA; 4 Department of Analytical Chemistry, Connecticut Agricultural Experiment Station, New Haven, CT 06504 USA

**Keywords:** Ingested engineered nanomaterial, iENM nanotoxicology, Nanosafety

## Abstract

**Background:**

Engineered nanomaterials (ENMs) are increasingly added to foods to improve their quality, sensory appeal, safety and shelf-life. Human exposure to these ingested ENMs (iENMS) is inevitable, yet little is known of their hazards. To assess potential hazards, efficient in vitro methodologies are needed to evaluate particle biokinetics and toxicity. These methodologies must account for interactions and transformations of iENMs in foods (food matrix effect) and in the gastrointestinal tract (GIT) that are likely to determine nano-biointeractions. Here we report the development and application of an integrated methodology consisting of three interconnected stages: 1) assessment of iENM-food interactions (food matrix effect) using model foods; 2) assessment of gastrointestinal transformations of the nano-enabled model foods using a three-stage GIT simulator; 3) assessment of iENMs biokinetics and cellular toxicity after exposure to simulated GIT conditions using a triculture cell model. As a case study, a model food (corn oil-in-water emulsion) was infused with Fe_2_O_3_ (Iron(III) oxide or ferric oxide) ENMs and processed using this three-stage integrated platform to study the impact of food matrix and GIT effects on nanoparticle biokinetics and cytotoxicity .

**Methods:**

A corn oil in phosphate buffer emulsion was prepared using a high speed blender and high pressure homogenizer. Iron oxide ENM was dispersed in water by sonication and combined with the food model. The resulting nano-enabled food was passed through a three stage (mouth, stomach and small intestine) GIT simulator. Size distributions of nano-enabled food model and digestae at each stage were analyzed by DLS and laser diffraction. TEM and confocal imaging were used to assess morphology of digestae at each phase. Dissolution of Fe2O3 ENM along the GIT was assessed by ICP-MS analysis of supernatants and pellets following centrifugation of digestae. An in vitro transwell triculture epithelial model was used to assess biokinetics and toxicity of ingested Fe_2_O_3_ ENM. Translocation of Fe_2_O_3_ ENM was determined by ICP-MS analysis of cell lysates and basolateral compartment fluid over time.

**Results:**

It was demonstrated that the interactions of iENMs with food and GIT components influenced nanoparticle fate and transport, biokinetics and toxicological profile. Large differences in particle size, charge, and morphology were observed in the model food with and without Fe_2_O_3_ and among digestae from different stages of the simulated GIT (mouth, stomach, and small intestine). Immunoflorescence and TEM imaging of the cell culture model revealed markers and morphology of small intestinal epithelium including enterocytes, goblet cells and M cells. Fe_2_O_3_ was not toxic at concentrations tested in the digesta. In biokinetics studies, translocation of Fe_2_O_3_ after 4 h was <1% and ~2% for digesta with and without serum, respectively, suggesting that use of serum proteins alters iENMs biokinetics and raises concerns about commonly-used approaches that neglect iENM – food-GIT interactions or dilute digestae in serum-containing media.

**Conclusions:**

We present a simple integrated methodology for studying the biokinetics and toxicology of iENMs, which takes into consideration nanoparticle-food-GIT interactions. The importance of food matrix and GIT effects on biointeractions was demonstrated, as well as the incorporation of these critical factors into a cellular toxicity screening model. Standardized food models still need to be developed and used to assess the effect of the food matrix effects on the fate and bioactivity of iENMs since commercial foods vary considerably in their compositions and structures.

**Electronic supplementary material:**

The online version of this article (10.1186/s12989-017-0221-5) contains supplementary material, which is available to authorized users.

## Background

Many foods contain organic or inorganic nanosize particles, which may be present naturally in the food itself [1, 2], be unintentionally generated during food processesing, or be introduced from the environment or packaging materials [3–7, 2]. Engineered nanomaterials (ENMs) are also often added to commercial food products intentionally, exploiting the unique or enhanced properties of nanoscale particles relative to their larger counterparts in order to improve food quality, sensory appeal, shelf life or safety [8–15]. For example, TiO_2_ is used to enhance color, texture and flavor [8, 16]; SiO_2_ is used as an anti-caking agent, and to clear beers and wines [9]; Fe_2_O_3_ is added as a food colorant; and ZnO is added to dietary supplements and breakfast cereals as a source of zinc [9]. In addition, inorganic ENMs are added to foods to provide specific flavors or colors, and are used in capsules and excipients for drugs and nutraceuticals [17–20]. Nanocellulose (NC) may be used in packaging to reduce bacterial growth and increase shelf life, as a source of dietary fiber, to stabilize emulsions and foams, to increase bulk, texture and appearance of baked goods, and to retain water in cooked meat products [14, 15, 21–23]. Conventional micron-sized forms of these materials have been approved for use in food by various regulatory authorities, and are generally regarded as safe (GRAS), without characterization or definition of particle dimensions. However, many of the approved GRAS materials also include a substantial component of nanosize particles. For example, food grade TiO_2_ (E171 – European designation) contains nano-scale particles [10], and nano-sized TiO_2_ was found in dietary supplements and food products [12]. Likewise, nano-sized SiO_2_ particles have been found in food grade silicon dioxide powders (E551), and in commercial foods and dietary supplements [10–12, 24].

The majority of nanotoxicological studies to date have focused on non-oral routes of exposure. Recently, interest in oral exposure to ENMs has been increasing, and growing numbers of both in vitro and in vivo studies of iENM biokinetics and toxicology are appearing in the literature [25–31]. To address the potential hazards of an increasing diversity of iENMs, and to promote a safer-by-design approach to iENMs development, we need to establish practical, standardized, and physiologically-relevant methodologies for evaluating the biokinetics and toxicology of iENMs using cellular and animal models. Most importantly, these methodologies must reproduce and account for the physicochemical transformations of iENMs that occur when they are incorporated into food products (food matrix effects) and as they pass through the GIT (gastrointestinal effects). While evidence continues to grow and show that such transformations can greatly impact the biokinetics and nano-biointeractions of iENMs, and alter the bioavailability and bioaccessibility of nutrients [31, 32], until recently they have been largely overlooked [3, 5]. In in vitro cellular studies, pristine iENMs are often mixed with culture media containing serum proteins and applied to cells to assess their bioactivity [31, 33]. It is well known that the use of serum protein and protein corona formation alters cell-nanoparticle interactions, including identification, and thus the biological effects of iENMs [34–36]. Furthermore, the food matrix-GIT property transformations of iENMs and their proper characterization are usually ignored in cellular studies [3].

**Fig. 1:**
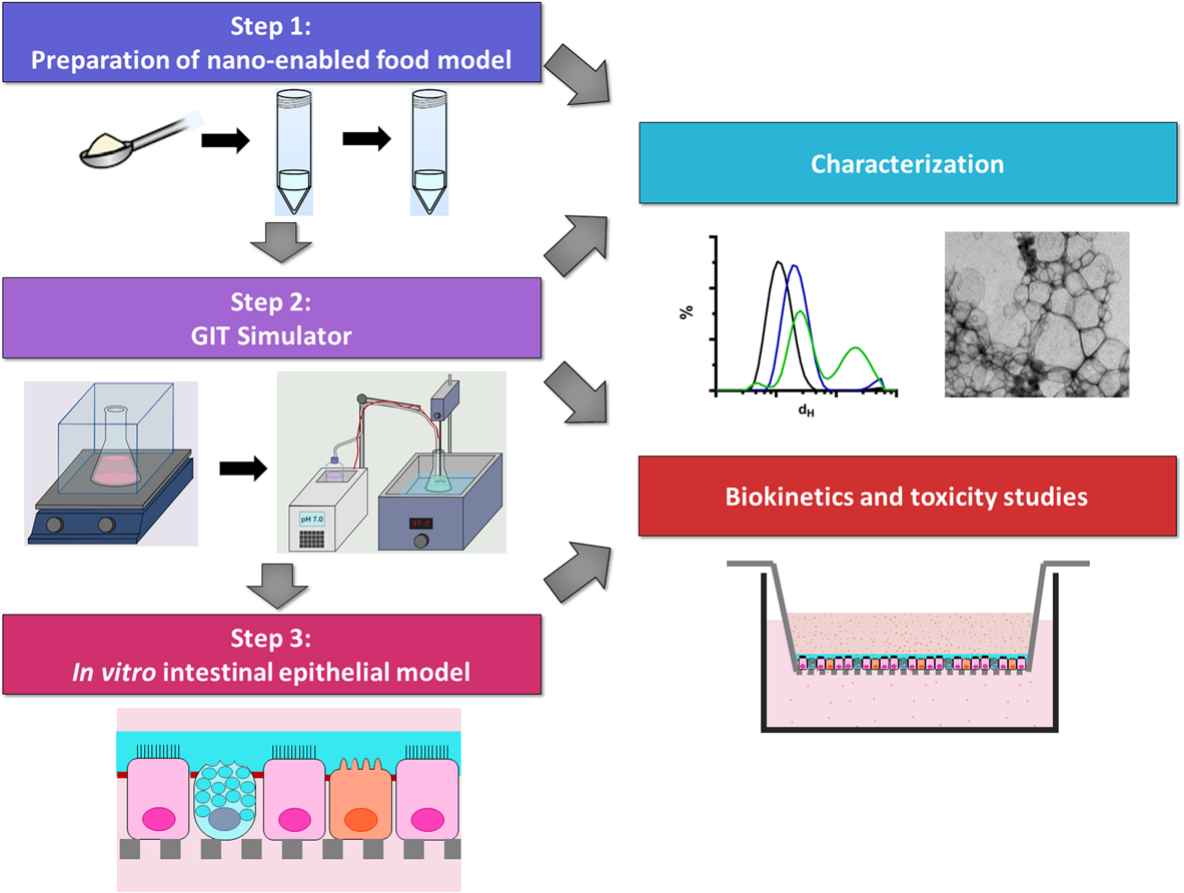
iENM in vitro studies overview: An overview of the integrated platform for studies of iENMs using simulated digestion of nano-enabled food model and biokinetics and toxicity studies using a triculture model of intestinal epithelium

Here, we present an integrated methodology for in vitro evaluation of iENM biokinetics and toxicity (Fig. 1). The approach consists of three modules: 1. Generation of nano-enabled food models and assessment of iENM-food interactions. 2. Simulated GIT digestion of nano-enabled food models, including mouth, stomach and small intestinal phases, with subsequent characterization of iENM transformations across the GIT, and 3. Development and utilization of an in vitro intestinal epithelial model, including cells representing enterocytes, mucus-secreting goblet cells, and microfold- or M-cells, suitable for in vitro assessment of biokinetics and toxicity of the iENMs [37–41]. Morphological and physicochemical characterization of iENMs to understand their transformations after incorporation in food and subsequent GIT digestion are crucial elements of this methodology. The efficacy of this integrated methodology was assessed in a case study using an oil-in-water emulsion food model in which Fe_2_O_3_ ENMs were incorporated.

## Methods

The integrated methodology detailed below provides a simple and efficient platform for the in vitro study of the biokinetics and toxicology of iENMs. The methodology consists of three interconnected modules (Fig. 1).

### Module 1: Assessment of iENM-food interactions (food matrix effect) using model foods

It is well known that in addition to the intrinsic properties of ENMs, the extrinsic properties and composition of the biological media as well, as the method of preparation of ENM suspensions, are critical determinants of the morphological, physicochemical and interfacial properties of the ENMs in suspension [42–46]. Likewise, one would expect that the intrinsic properties of the iENMs, the composition and properties of the food matrix and the method whereby the iENM is incorporated into the food model will play a major role in determining their subsequent nano-biointeractions.

#### Selection of food model

For iENM studies, commonly used food models used in drug and nutrient delivery stydies can be utilized to simulate an array of dietary conditions. It is well known that the bioavailability and biokinetics of drugs and nutrients are strongly influenced by the selection of food model. For example, milk significantly delays tablet dissolution [47], but increases solubility of many drugs relative to a fasting media [48, 49], and binding of drugs to milk components often increases with fat content [50]. Although the most physiologically accurate food state is a homogenized meal equivalent to that which might be consumed with a drug or nutraceutical [51], analyses of drugs, nutraceuticals or iENMs in such a complex media, and at later stages of digestion, may be difficult. Simpler formulations, including EnsurePlus® and whole milk, have physicochemical properties resembling the HSS-FDA recommended standard meal for evaluation of drug bioavailability [52], and have been used in many studies to evaluate analyte dissolution and bioavailability [48, 49, 53–55].

In our iENM case study, a simple model food consisting of an oil-in-water emulsion stabilized by a protein emulsifier was selected. This and similar food models have been used in many studies for in vitro assessment of the fate and bioavailability of micronutrients [56–61]. Because of its simplicity, the model allows relatively easy characterization and analysis of the iENM within the food and throughout the simulated GIT processes. Specifically, we employed a 2% corn oil-in-water emulsion (97.8 wt% phosphate buffer) stabilized with 0.2 wt% whey protein. In addition to the relative ease of analysis in such emulsions and their digestae, the composition and structure of the system can be easily modified to study their effects on gastrointestinal fate, bioavailability and biokinetics, of adding or altering various components (e.g. changing fat type or content, adding sugars, starch, protein, etc.). Although the integrated methodology presented here can be followed in principle with any food model, more complex food models may present additional challenges, particularly for investigation of iENM-food-GIT interactions, which will require further adaptation of our methodology and development of characterization techniques for such complex biological media. We are currently developing and testing standardized complex food models similar to those used to study dissolution, bioavailability and biokinetics of pharmaceuticals that can be used in cellular studies of iENMs utilizing the integrated methodology presented here.

#### Preparation of nano-enabled food model to assess iENM-food matrix interactions

While there are numerous possible ways of mixing iENM with the model food described above, in order to provide a reproducible standardized methodology, we adapted a method that has been proven to provide maximally stable ENM dispersions in physiological media for cellular studies. As detailed elsewhere [42, 62], this method involves first creating a suspension of the ENM powder in water by sonicating above a known critical energy (DSE_cr_), followed by subsequent dilution to the desired concentration in media. Thus, in the method adapted for studying iENMs, we first created a dispersion of the iENM (Fe_2_O_3_ in our case study) in water. The aqueous iENM dispersion was then mixed with the model food, which in our case study, was an oil-in-water nanoemulsion containing 2.0 wt% corn oil, 0.2% whey protein, and 97.8% of phosphate buffer (5 mM, pH 7.0).

**Fig. 2:**
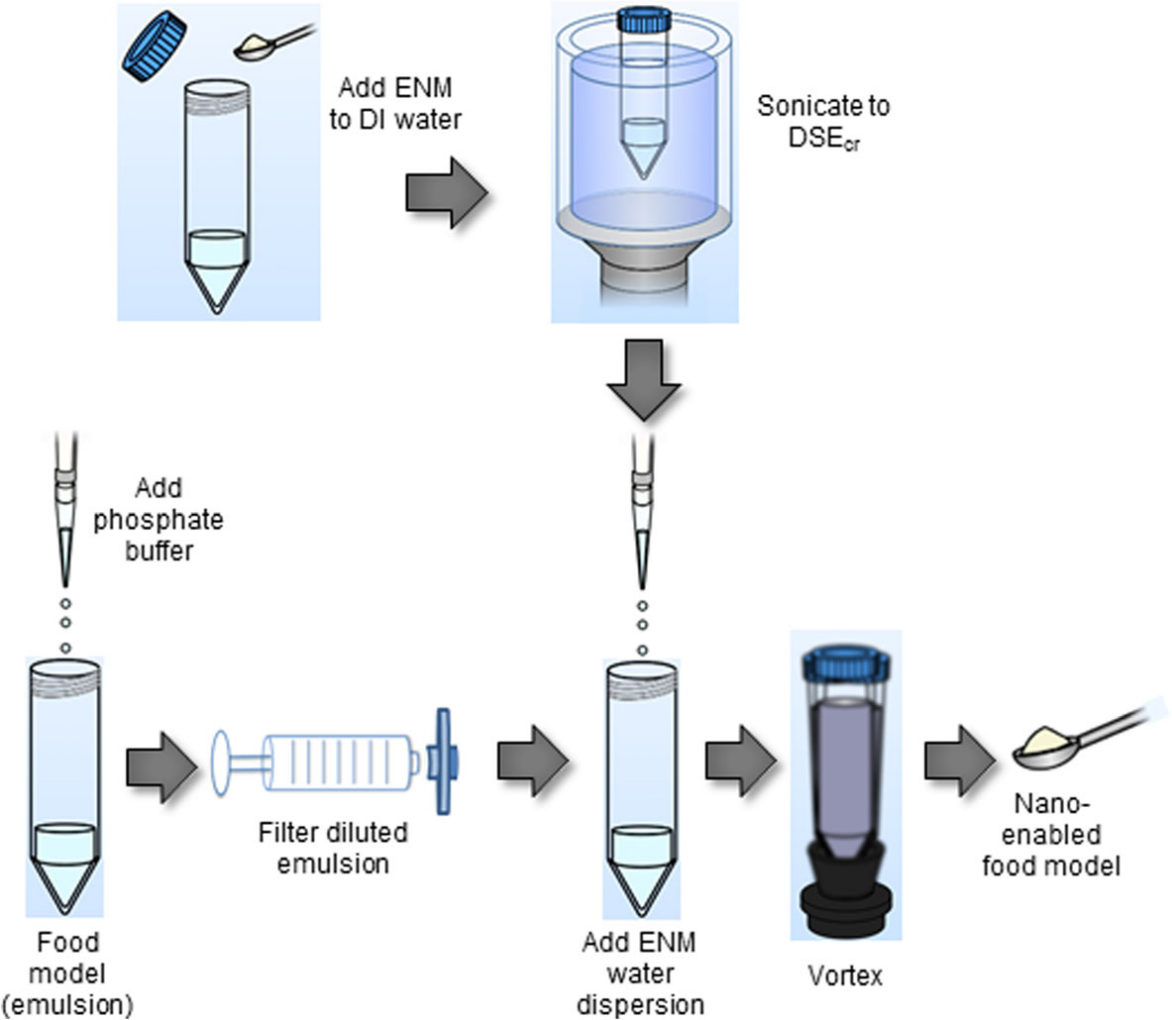
Preparation of nano-enabled food model: Sequence of steps used to add ENM to an oil/water

A detailed schematic of the methodology for creating the nano-enabled model food presented here is given in Fig. 2. The detailed protocols employed for preparation of the Fe_2_O_3_ water dispersion, stock corn oil nanoemulsions and Fe_2_O_3_ iENM nano-enabled emulsion food model are provided in the Additional file 1.

### Module 2: Development and utilization of a three stage GIT simulator including mouth, stomach and small intestinal phases for the digestion of the nano-enabled food model

A bench top, three stage GIT simulator, including mouth, stomach and small intestinal phases, for studying the gastrointestinal fate of the nano-enabled model food was developed based on models previously developed by the authors [56, 57].

**Fig. 3:**
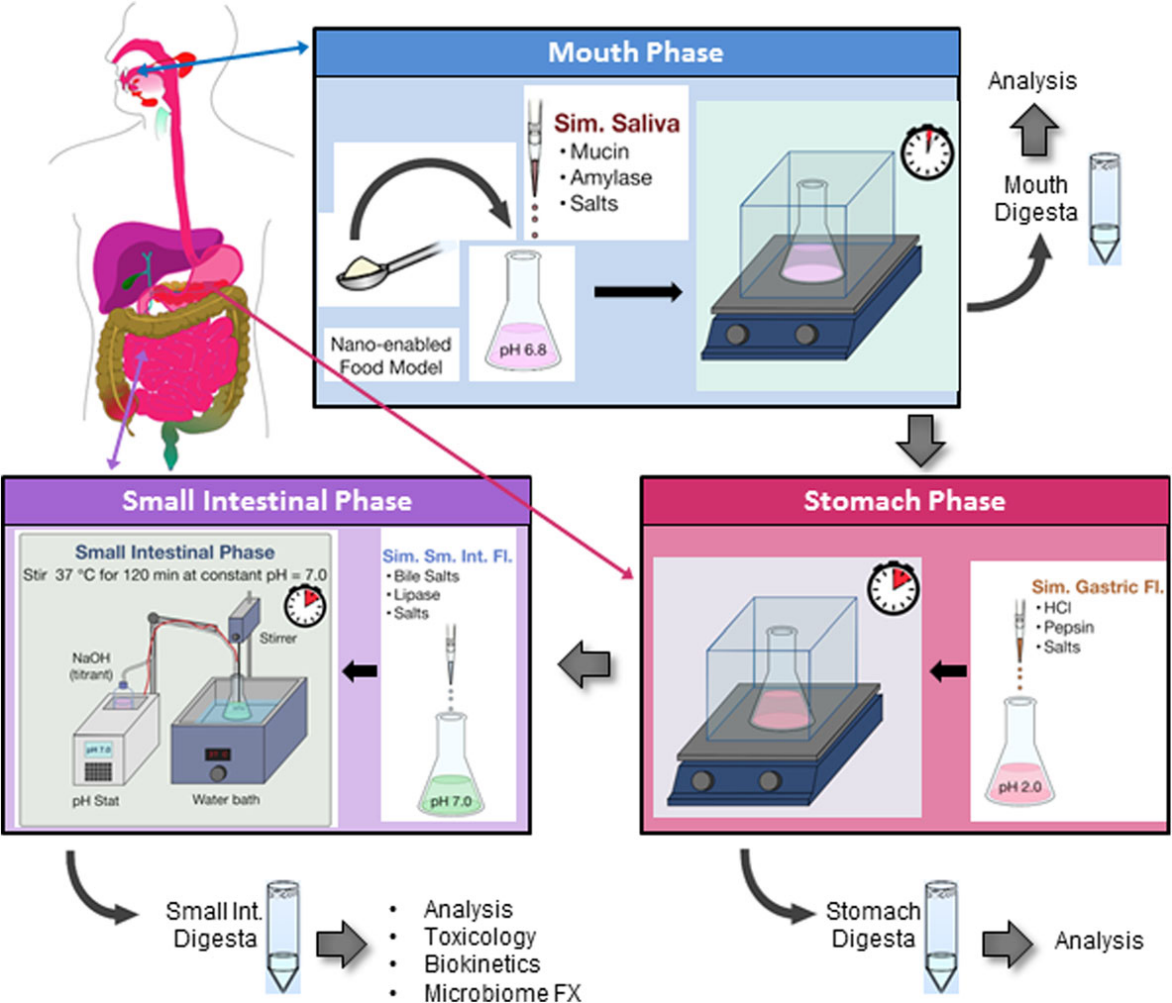
GIT Simulator. Simulated digestion of nano-enabled food consists of (clockwise from top) a mouth, stomach and small intestinal phase

The GIT simulator process employed in this study is illustrated schematically in Fig. 3. Briefly, in the mouth phase of the GIT simulator, the nano-enabled food was mixed and incubated together with a simulated saliva fluid for 2 min. The resultant mouth digesta (“bolus”) was then combined and incubated with a simulated gastric fluid for 2 h to represent the stomach digestion phase. In the small intestinal phase, the stomach digesta (“chyme”) was combined with bile salts and proteins simulating the intestinal fluid, and incubated for 2 h at a pH of 7.0 which is maintained constant by use of a pH Stat titration device. It should be pointed out that the pH in vivo varies across to small intestine, from about 6.0 in the duodenum to 7.4 in the terminal ileum [63], and a more accurate system would require adjusting the pH throughout the small intestinal digestion accordingly. We chose to avoid this additional complication by using an approximate average pH of 7.0 across the small intestine, as commonly used in the food science literature for intestinal digestion [56, 57]. Maintaining a constant pH of 7.0 also provides the advantage of allowing calculation of fatty acid hydrolysis from triglycerides, for example, from the amount of titrant required to maintain the constant pH. Details of the materials, preparation of simulated saliva, gastric fluid and small intestinal fluid, and the performance of the simulated GIT model are provided in the Additional file 1.

### Module 3: Development and morphological characterization of an in vitro intestinal epithelial model for iENM biokinetics and toxicity studies

A number of in vitro models have been used to study the toxicity and biokinetics of pharmaceuticals and chemicals in the GIT. The most commonly used model employs Caco-2 cells (immortal human colonic epithelial) cells, which after culture for 2–3 weeks differentiate into cells with markers and morphological characteristics of small intestinal epithelial enterocytes [64–66]. While this may be a reasonable choice for many situations, the epithelium of the small intestine is more complex, and in order to more accurately emulate this structure, a variety of modifications have been added. The intestinal mucosa is normally protected by a layer of mucus produced by both goblet cells and submucosal glands (Brunner’s glands, limited mostly to the duodenum) [67]. It is therefore appropriate to modify the in vitro model to include mucus secreting cells. To this end, HT29-MTX cells, an immortal human cell line that resembles intestinal goblet cells and secretes mucus, is often co-cultured with Caco-2 cells [66–69]. Finally, in the Peyer’s patches and other lymphoid-associated epithelium of the small intestine, specialized cells called Microfold- or M-cells are present. These cells engulf and translocate samples of the contents of the intestinal lumen to lymphocytes in the submucosa below, thereby providing continuous antigenic surveillance of the intestinal contents [33]. It has also recently been shown that M-cells can play an important role in translocation of iENMs in in vitro intestinal epithelial models [33]. It has previously been shown that differentiated Caco-2 cells can be induced by factors released from another cell line, Raji B (a human B lymphocyte) to differentiate into cells resembling M-cells [70, 71]. Thus, when Raji B cells are added to the basolateral compartment of a transwell system in which matured caco-2 cells reside on the transwell membrane above, some of the Caco-2 cells are induced to differentiate into M-like cells. The complete hybrid triculture model utilized in our methodology, illustrated in Fig. 5a, has previously been described and characterized and includes cells with morphology and markers consistent with the three primary cells of the intestinal epithelium: enterocytes, goblet cells and M-cells [37–41]. Because it represents a reasonably realistic hybrid model of the complete intestinal epithelium, this model was adopted for the proposed integrated methodology. Specifically, we employed the protocol reported by Mahler et al. [37] for development of our triculture system. Such a physiologically relevant model is well suited to the study of biokinetics and intestinal toxicity of iENMs. However other similar advanced models could also be used.

Details of the methods employed for development, characterization and validation of the triculture model, including protocols for creating the system, measurement of transepithelial electrical resistance (TEER), immunofluorescence staining and imaging for morphological characterization and TEM characterization are provided in Additional file 1.

#### Biokinetics and toxicity experiments

As mentioned in the introduction, it is quite common in cellular toxicity studies of iENMs to use “pristine” nanomaterials exposed to cell culture models to measure their bioactivity, thus ignoring iENM-food-GIT interactions and transformations that may have a significant effect. In our proposed modular approach, the iENMs are exposed to the food matrix and simulated GIT before being applied to a tri-culture cellular model, which should provide a more accurate representation of their biological fate.

In more detail, the nano-enabled model food (Module 1) is exposed to the GIT simulator (Module 2) and then applied to the transwell triculture model of the intestinal epithelium (Module 3) to determined the biokinetics and toxicity of the iENMs. The experimental design for these studies is depicted in Additional file 1: Figure S1.

##### Fine tuning and troubleshooting experiments to eliminate toxicity of small intestine digest

It is worth noting that in initial experiments, we observed that the small intestinal digesta without ENMs (control) was highly toxic to the triculture cells, and therefore unsuitable for iENM biokinetics or toxicity studies. Based on the osmolarity calculated for each solution used in the simulated digestion (Additional file 1: Table S2), the osmolarity of that digesta was calculated to be 654 mOsm/L; which is more than double isotonic (~280–290 mOsm/L). Since exposure of cells to such a hypertonic solution would cause a loss of water leading to cell shrinkage, crenation, and likely cell injury and death, we hypothesized that this was a potential cause of the observed toxicity. Another potential source of cell injury in the digesta was bile salts, which were present in our initial small intestinal digesta at a concentration of ~10 mM. Bile salts play a number of important roles in food digestion by facilitating the emulsification of ingested lipids, aiding the adsorption of lipase to lipid droplet surfaces, and solubilizing and transporting lipid digestion products and hydrophobic bioactive agents. However, because of their detergent properties they can also be toxic to cells when present at sufficiently high levels. Indeed, bile salts have been shown to be capable of causing injury to tissues throughout the GIT [72–74]. In a number of fine-tuning experiments, the toxicity of the final small intestine phase digesta was reduced by adding sufficient phosphate buffer (5 mM, pH 7.0) to lower the osmolarity (280 mOsm/L) and bile salt concentration (~4 mM) to lower but still physiologically relevant levels.

Finally, in order to ensure adequate nutrient delivery to cells for proper function and viability, it was also necessary to dilute the final digesta to 1:3 with cell culture media without the presence of any serum proteins (i.e. FBS) to ensure the required nutrient delivery. The absence of serum proteins is important because they would likely alter the agglomeration state and protein corona of the iENMs, both of which are critical determinants of nano-biointeractions [34–36, 75, 76]. Previous studies have often diluted digesta 20-fold or more in media containing serum [29, 30] which may have an effect on the bioactivity of iENMs.

The final simulated GIT digestion protocol described briefly in the three modules above is detailed in the Additional file 1 and reflects the trouble-shooting and fine tuning described above. Biokinetics and iENM toxicity studies for the Fe_2_O_3_ case study were performed by applying a 1:3 dilution of the final small intestinal digesta in serum-free DMEM to the apical compartment of the transwell triculture, and incubating for the desired time period. The initial concentration of Fe_2_O_3_ in the nano-enabled food model was either 0.05 or 0.1% by weight for biokinetics experiments, and 0.1% by weight for toxicity experiments. These concentrations were based on the 0.1% by weight maximum concentration in food allowed by the FDA (https://www.accessdata.fda.gov/scripts/cdrh/cfdocs/cfcfr/CFRSearch.cfm?fr=73.200). Detailed protocols for biokinetics and toxicity experiments are also provided in the Additional file 1.

#### Colloidal characterization of ENM dispersions, emulsions and digestae across the GIT

Key to understanding the iENM toxicological properties and biokinetics is information about the physicochemical transformations that the iENMs undergo when they are incorporated into foods, and as they pass through the GIT. Unfortunately, because of the complex composition of even the simplest foods, this task is as challenging as it is important. We have employed a number of state of the art analytical methods for the characterization of iENM-food-GIT interactions and property transformations. However, it is worth noting that additional methods can also be utilized to supplement these approaches so as to more thoroughly characterize the complex interactions involved [3, 77].

For our case study, the particle size distribution was characterized across the iENM-food-GIT continuum using both dynamic light scattering (DLS) and laser-diffraction analysis. Further morphological characterization was performed by staining the lipid phase with Nile red (a fluorescent lipophyic dye) and and examining the samples with fluorescence confocal microscopy. In addition, TEM was used to examine the morphology of the model food emulsion and digestae across the simulated GIT.

Another important transformation that must be considered for iENMs is dissolution, particularly since these materials must travel through the highly acidic environment of the stomach. Accordingly, dissolution of the Fe_2_O_3_ ENMs was assessed in the model food as well as after exposure to each phase of the simulated GIT.

Details about the methods used for emulsion and digestae particle size distribution characterization, fluorescent staining and confocal microscopy, TEM imaging, assessment of dissolution, and protein corona analysis are provided in the Additional file 1.

#### Pristine ENM synthesis and characterization

##### ENM synthesis

The ENMs used in this study were synthesized and characterized as part of the HSPH-NIEHS Reference ENM repository established at Harvard as part of the Nantional Insititute of Environmental Health Sciences (NIEHS) Nanotechnology Health Implication Research (NHIR) Consortium. Details of methods for the synthesis of the Fe_2_O_3_ and Au ENMs used to assess the role of serum in the diluting media on protein corona are provided in the Additional file 1.

##### Pristine ENM characterization

Details about the analytical methods used for characterization of the pristine ENMs, including Brunauer–Emmett–Teller (BET) analysis to determine Specific surface area (*SSA*), and calculation of primary particle diameter (*d*_BET_), X-Ray Diffraction (XRD) analysis to estimate crystal dimensions (*d*_XRD_), TEM imaging for morphological characterization, and endotoxin analysis are provided in the Additional file 1.

## Results

### Characterization of pristine ENMs

The results from the characterization of the Fe_2_O_3_ ENM powder are reported in Table 1. TEM images of pristine Fe_2_O_3_ ENMs are shown in Additional file 1: Figure S3. Endotoxin levels the Fe_2_O_3_ ENMs was below the limit of detection (0.005 EU/ml) at 10 μg/ml, with no evidence of assay inhibition in samples spiked with 0.5 EU/ml of endotoxin standard.

**Table 1:** Characterization of surface area and mean particle diameter of pristine Fe_2_O_3_ ENMs used in this study

Material	SSA (m^2^ g^−1^)	*d*_BET_ (nm)	*d*_XRD_ (nm)
VENGES Fe_2_O_3_	54.7±3.7	22.5±1.5	24.5

*SSA* (specific surface area) by nitrogen adsorption/Brunauer-Emmett-Teller (BET) method, *d*_BET_, primary particle diameter determined from *SSA*, *d*_XRD_ particle diameter as determined by X-ray diffraction, *d*_TEM_ particle diameter as determined by TEM image analysis, *d*_DLS_ particle diameter as determined by DLS

### Colloidal characterization of food model with and without Fe_2_O_3_ ENM and digestae across the GIT

**Fig. 4:**
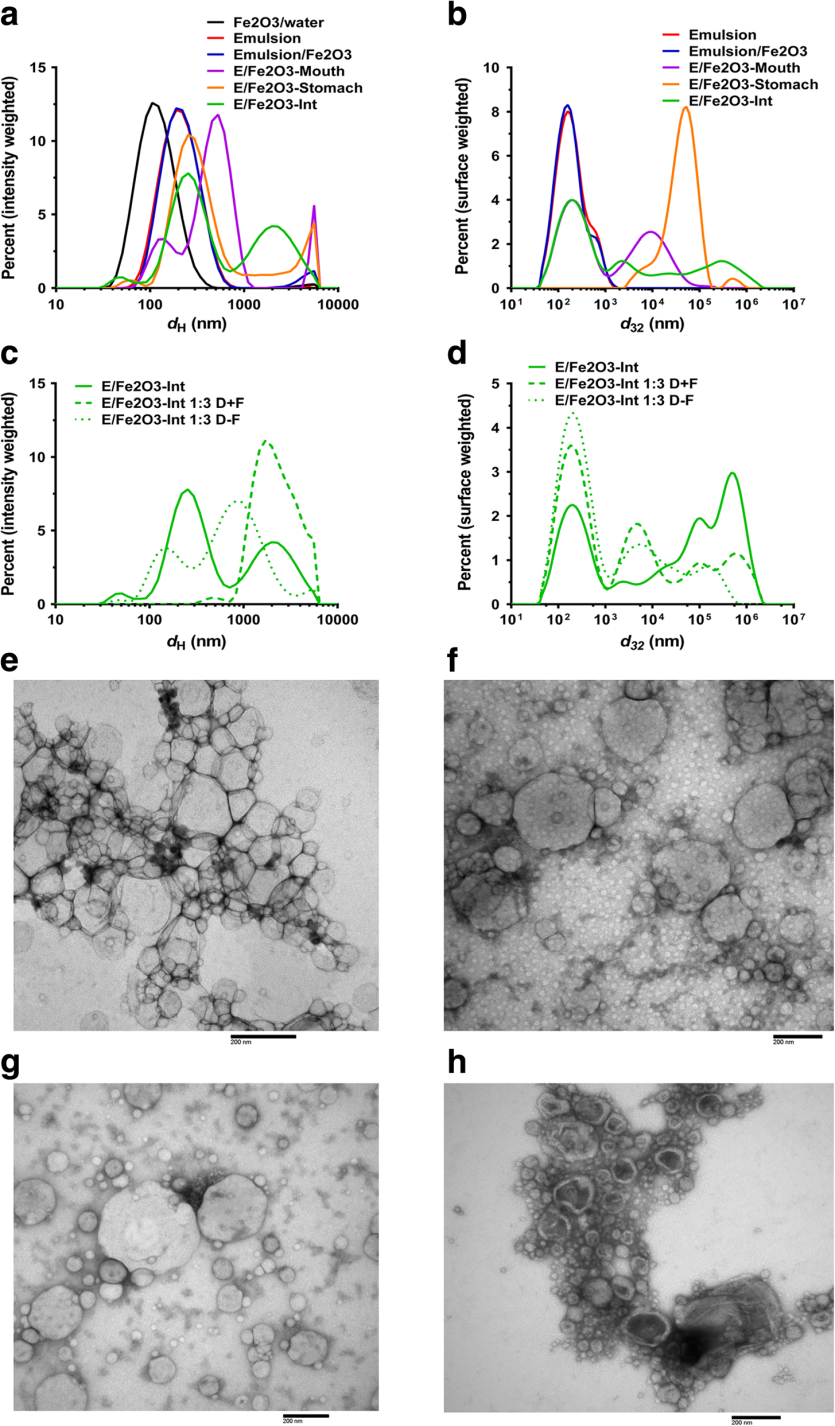
Characterization of nano-enabled food model and digestae: size distributions. **a** Size distributions of emulsion and nano-enabled emulsion digestae by DLS (hydrodynamic diameter d_H_, intensity-weighted). **b** Size distributions of emulsion and nano-enabled emulsion digestae by laser diffraction (d_32_, surface-weighted). distribution of emulsion and digestae by DLS. **c** Size distributions by DLS of final small intestinal digesta and its dilutions in media with and without FBS. **d** Size distributions by laser diffraction of final small intestinal digesta and its dilutions in media with and without FBS. **e** TEM image of nano-enabled (0.1 wt% Fe_2_O_3_ ENM) food model. **f** TEM of mouth digesta. **g** TEM of stomach digesta. **h** TEM of small intestinal digesta

Colloidal characterization, including particle size distributions by DLS and laser diffraction of the food model emulsion with and without ENM before digestion, after each phase of simulated digestion, and after dilution of the final intestinal digestae with DMEM culture media with and withot FBS are summarized in Fig. 4 and Table 2.

**Table 2:** Colloidal characterization of ENM used in this study

Sample	*d*_H_ (nm)	*D*_32_ (nm)	PdI	ζ (mV)	σ (mS/cm)	pH
Fe_2_O_3_ in water	108.3 ± 1.1	–	0.199 ± 0.007	−27.2 ± 0.4	0.03 ± 0.00	6.05
Emulsion	191.7 ± 2.2	138 ± 1	0.190 ± 0.019	−33.6 ± 1.3	0.81 ± 0.00	6.97
Emulsion + Fe_2_O_3_	200.7 ± 4.1	132 ± 3	0.243 ± 0.007	−36.3 ± 0.6	0.80 ± 0.01	6.94
Mouth digesta	365.4 ± 20.3	295 ± 9	0.489 ± 0.096	−23.0 ± 0.8	1.04 ± 0.03	6.66
Mouth digesta + Fe_2_O_3_	472.3 ± 4.3	270 ± 8	0.768 ± 0.008	−23.7 ± 1.1	1.03 ± 0.3	6.76
Stomach digesta	272.4 ± 5.9	29,967 ± 1254	0.354 ± 0.052	0.7 ± 0.5	1.49 ± 0.07	1.64
Stomach digesta + Fe_2_O_3_	332.2 ± 17.3	25,490 ± 550	0.541 ± 0.073	1.4 ± 0.2	1.40 ± 0.14	1.52
Sm. Int digesta	227.5 ± 1.7	1175 ± 182	0.322 ± 0.030	−55.2 ± 3.7	1.05 ± 0.03	7.02
Sm. Int digesta + Fe_2_O_3_	335.5 ± 0.7	491 ± 52	0.513 ± 0.015	−47.6 ± 1.7	1.08 ± 0.04	6.98
Sm. Int. digesta 1:3 D + F	1736.7 ± 160.9	405 ± 40	0.390 ± 0.130	−16.5 ± 1.1	2.23 ± 0.10	7.13
Sm. Int. digesta + Fe_2_O_3_, 1:3 D + F	1867.7 ± 144.3	336 ± 62	0.373 ± 0.023	−16.3 ± 1.6	2.29 ± 0.15	7.03
Sm. Int digesta 1:3 D-F	242.4 ± 1.7	347 ± 8	0.322 ± 0.040	−27.2 ± 1.4	2.17 ± 0.10	7.07
Sm. Int digesta + Fe_2_O_3_,1:3 D-F	394.2 ± 2.5	307 ± 52	0.536 ± 0.012	−24.9 ± 3.2	2.35 ± 0.14	7.10

*d*_H_ intensity-weighted mean hydrodynamic diameter by DLS, *D*_32_ surface-weighted mean diameter by laser diffraction, *PdI* polydispersity index, *ζ* zeta potential, *σ* specific conductance

The mean particle dimensions determined by DLS (intensity-weighted mean hydrodynamic diameter, *d*_H_) and laser diffraction (surface area-weighted diameter, *D*_32_) were similar for food emulsions in the absence and presence of Fe_2_O_3_ nanoparticles: *d*_H_ = 191.7 and 200.7 nm, and *D*_32_ = 138 and 132 nm for emulsion alone and for emulsion + ENMs, respectively. The particle size distribution was monodisperse for both the emulsion alone and for the emulsion + ENMs, with a slightly greater polydispersity for the mixed system (PdI = 0.243) than for the emulsion alone (PdI = 0.190). This result indicates that the proposed protocol can generate fairly monodispersed nano-enabled model foods for cellular biokinetic and toxicity studies, which would facilitate subsequent data interpretation.

After exposure to the mouth phase, the particle size distributions of the digestae both with and without ENMs were multimodal, and the mean particle sizes increased. Substantial differences were observed in both the particle size distribution and the mean *d*_H_ values between mouth digesta with and without ENMs (*d*_H_ = 365.4 and 472.3 nm for emulsion alone and emulsion + ENM, respectively), although a smaller difference was observed by laser diffraction analysis (*D*_32_ = 295 and 270 nm for emulsion alone and emulsion + ENM, respectively). Polydispersity was also substantially higher in the mouth digesta with ENM (PdI = 0.768) than without (PdI = 0.489). It is also notable that for mouth digesta as well as digestae from later steps, the mean particle sizes and distributions obtained by DLS differed substantially from those obtained by laser diffraction. This is likely a result of the difference in weighting (scattering intensity-weighting for DLS vs. surface area-weighting for laser diffraction), and, for suspensions containing larger particles, a result of the upper limit of ~2 μm for analysis by DLS vs. up to 1000 μm for laser diffraction. These differences are particularly pronounced in the stomach phase digestae where very large size species are present. This result highlights the importance of utilizing an appropriate analytical method for measuring particle size charateristics.

Following exposure to the stomach phase, the mean *d*_H_ (DLS) was 272.4 and 332.2 nm for digestae with and without ENM, respectively, and *D*_32_ (laser diffraction) was 30,000 and 25,500 nm for digestae with and without ENM. Although the mean particle sizes of digesta with and without ENM for each measurement method were roughly similar, the PdI was somewhat greater for stomach digestae with ENM (0.354) than without (0.541), and the particle size distributions of stomach digestae with and without ENM were clearly dissimilar.

Following exposure to the small intestinal phase, both methods revealed appreciable differences between digestae with and without ENM (*d*_H_ = 227.5 and 335.5 nm, and *D*_32_ = 1175 and 491 nm for digesta without and with ENM, respectively). Likewise, the size distributions differed considerably, and PdI was greater for digesta with ENM (0.513) than without ENM (0.322).

As noted above in the methods section, in order to utilize the small intestinal digestae to expose cells for biokinetics experiments using the triculture cell model, it was necessary to dilute it 1:3 in cell culture media (DMEM) to avoid damaging the cells and to provide the necessary nutrients to the cells over the time of the exposure. Moreover, it was noted that it is important that such a dilution has as little effect as possible on the protein corona and interfacial properties of the ENMs, and we hypothesized that the addition of FBS to the diluting media would substantially alter these properties and should thus be avoided. This hypothesis was borne out in part by the results of particle size characterization of small intestinal digestae diluted in DMEM with and without FBS. Mean particle sizes obtained by DLS for digestae diluted in DMEM + FBS (*d*_H_ = 1740 and 1870 nm for dilutions of digestae without and with Fe_2_O_3_ ENM, respecitively) were strikingly different from those obtained for dilutions without FBS (*d*_H_ = 242] and 394 nm for dilutions of digestae without and with Fe_2_O_3_ ENM, respecitively). More modest differences were observed by laser diffraction (*D*_32_ = 405 and 336 nm for dilutions in DMEM + FBS of digestae without and with Fe_2_O_3_ ENM, respectively, and *D*_32_ = 347 and 307 nm for dilutions in DMEM without FBS of digestae without and with Fe_2_O_3_ ENM, respectively). It is obvious that the presence of serum proteins resulted in ENM agglomeration and corona formation, a phenomenon known in cellular anotoxicology research [42, 44].

Furthermore, surface charge measurements (zeta-potential, ζ) of the model food emulsion and digestae throughout the GIT (Table 2), revealed moderately negative charge for emulsion without and with Fe_2_O_3_ ENM (−33.6 and −36.3 mV, respectively), as well as the mouth phase digesta (−23.0 and −23.7 mV without and with Fe_2_O_3_ ENM, respectively). In contrast, digestae from the stomach phase had a slightly positive charge (+0.7 and +1.4 mV for stomach digestae without and with Fe_2_O_3_ ENM, respectively). This reversal may be due to protonation of weak acid groups at the low pH (~1.5) of the stomach digestion model. Following small intestinal digestion (pH = 7.0), the surface charge was more strongly negative than in the original emulsion and mouth phase (−55.2 and −47.6 mV for digestae without and with ENM, respectively). Importantly, dilution of the small intestinal digestae in DMEM with FBS decreased the magnitude of this negative charge more markedly (−16.6 and −16.3 mV without and with ENM) than did dilution in DMEM alone (−27.2 and −24.9 mV without and with ENM), further underscoring the importance of avoiding the addition of serum protein to the dilution, whch is currently the standard practice in the field.

### TEM and confocal fluorescence imaging of emulsions and digestae throughout GIT

TEM images of emulsion and digestae throughout the simulated GIT are shown in Fig. 4. In these images, it is clear that the size distribution, aggregation state, and morphology of the lipid droplets and associated Fe_2_O_3_ particles changed dramatically between the emulsion and mouth phase, and between the mouth, stomach and small intestinal phases. In the original emulsion with Fe_2_O_3_, lipid droplets are smooth and round to polygonal in shape, forming aggregates containing large numbers of droplets of varying size. Individual and small groups of Fe_2_O_3_ particles can be seen interspersed among the droplets. In the mouth phase, irregular shaped agglomerates of various sizes were observed, which contained a mixture of lipid droplets and Fe_2_O_3_ particles. The morphology and agglomeration of the droplets in the stomach phase was similar to that observed in the mouth phase. However, no Fe_2_O_3_ particles were observed in the images of the stomach digesta. Finally, in the small intestinal phase, the digesta contained large agglomerates containing particles of varying size and morphology. Notably, some of the particles present appear to be deflated, which is likely due to the action of lipase digestion of the triacylglycerols to monoacylglycerols and free fatty acids.

Confocal microscopy and TEM images of the model food and digesta formed throughout the GIT are shown in Additional file 1: Figure S4. The size and morphology of the particles observed by these methods are consistent with the DLS and laser diffraction size analysis described above and point to important transformations across the IENM-food-GIT continuum.

### Validation of the triculture intestinal epithelial model

**Fig. 5:**
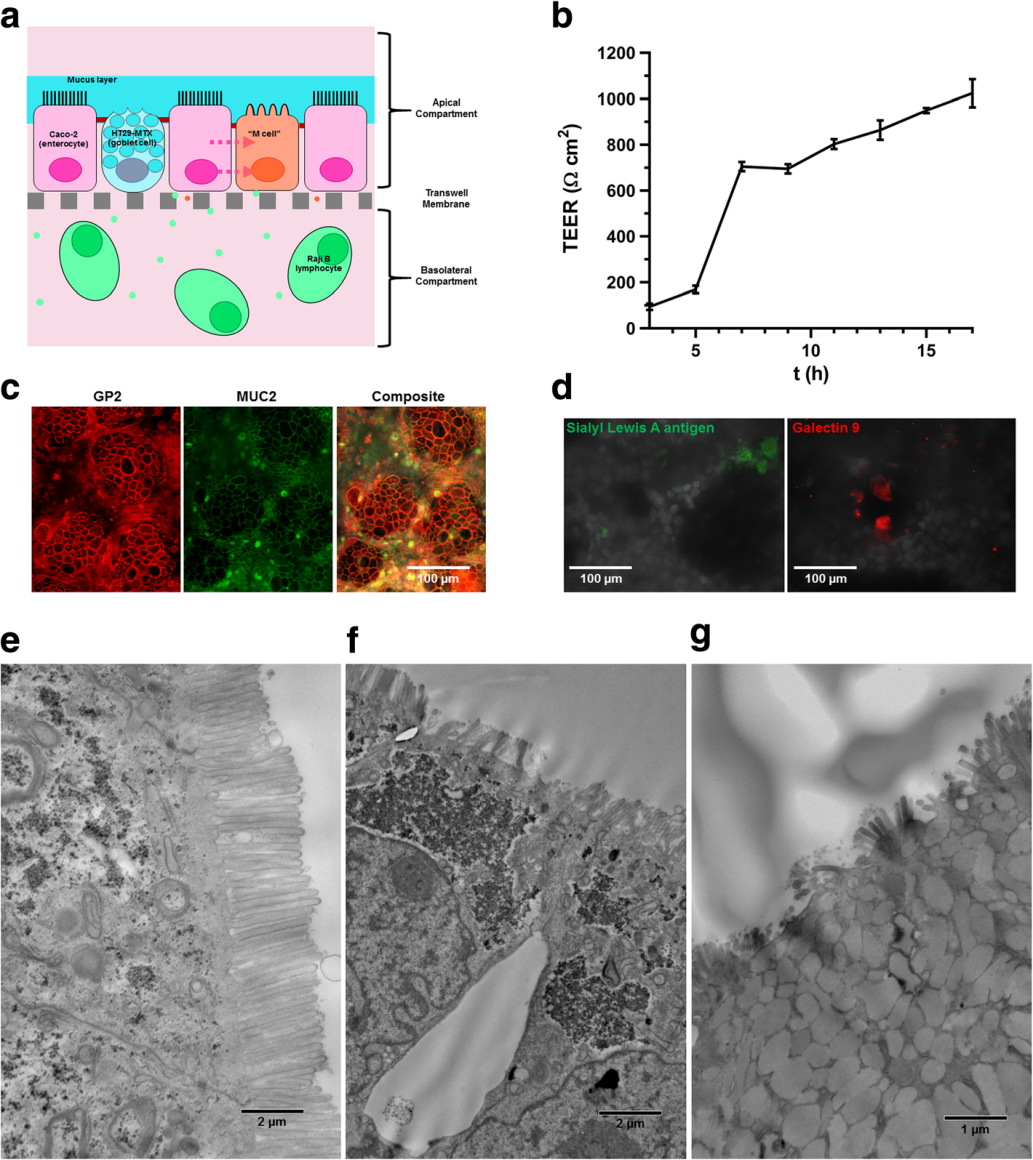
In vitro triculture intestinal epithelial cell culture model characterization. **a** triculture model schematic. **b** TEER over time. **c** Mucin and GP2 immunostain. **d** Sialyl Lewis A antigen and Galectin 9 staining. **d** Sialyl Lewis A antigen and Galectin 9 staining. **e** TEM of enterocyte. **f** TEM of M-cell. **g** TEM of goblet cell

TEER measurements confirmed that the transwell cultures were continuous and intact, with TEER values exceeding 900 Ω-cm^2^ in fully mature triculture wells (Fig. 5). Immunostaining and confocal fluorescence imaging revealed the presence of mucin (Muc-2) in the cells surrounding nests of enterocytes. In addition, on gross appearance the monolayers were observed to be coated with a thick layer of mucus. A small fraction of cells (<1%) appeared within nests of enterocytes exhibiting one of several reported markers of M-cells, including GP-2, Sialyl-Lewis A antigen and Galectin-9 (Fig. 5c and d). TEM imaging of the triculture model revealed a single monolayer of cells, including cells with the morphological characteristics consistent with all three constituent cells of small intestinal epithelium: enterocytes with prominent and well-defined microvilli; goblet-like cells packed with mucus-filled vacuoles; and, M-cells with blunted and fused microvilli (Fig. 5e, f and g).

It is also worth noting that as discussed in the methods section under ‘troubleshooting’, the digesta (with or without ENM) created using the original GIT simulator protocol were highly toxic to the triculture cells. Adjustment of osmolarity in the intestinal phase reduced but did not eliminate toxicity. A 1:3 dilution of the digestae with complete DMEM media (with or without FBS) was sufficient to maintain cell viability. The effects of these adjustments to our proposed digestion protocol on cell viability were evaluated by live/dead fluorescent staining, as well as toxicity of diluted digestae with and without Fe_2_O_3_ ENM measured by LDH assay, and are shown in Additional file 1: Figure S5.

Furthermore, as mentioned above, it is necessary to dilute the final small intestinal digestae in media priot to application to the cell culture model in order to provide the necessary nutrients and maintain cell viability. However, in order to minimize the effects on the interfacial properties of the iENMs that may affect nano-bioninteractions, it is important to avoid addition of proteins, specifically serum, to the diluting media.

### Dissolution of Fe_2_O_3_ in model food emulsion and throughout the simulated GIT digestion

ICP-MS analysis of the whole digestae and fractions obtained from high speed centrifugation (fat, micelle and pellet) revealed minimal dissolution of Fe_2_O_3_ at all three stages of digestion, with a maximum of 2% total dissolution occurring in the small intestinal digesta (Additional file 1: Figure S6). In the mouth, stomach and small intestinal digestae, equal amounts of Fe_2_O_3_ were dissolved in the fat and micelle (supernatant) fractions. Dissolution decreased to <0.25% after the small intestinal digesta was diluted (1:3) in DMEM (without FBS) prior its use to expose the triculture gut epithelia model.

### Biokinetics and toxicity of digested Fe_2_O_3_ ENMs

**Fig. 6:**
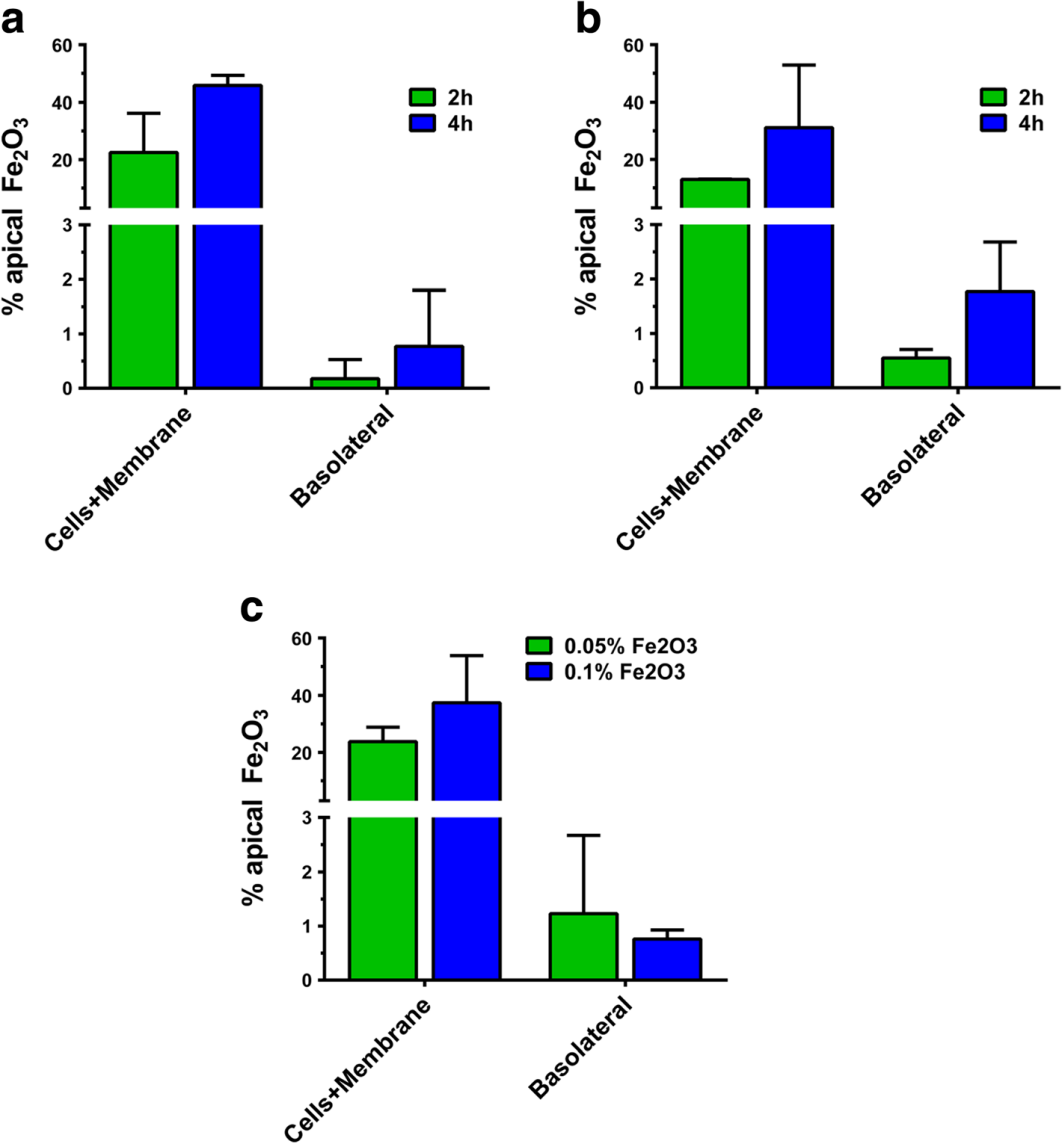
Biokinetics study results. Percent of applied (apical compartment) Fe_2_O_3_ in cells + membrane and basolateral compartments following incubation of transwell triculture inserts for the times indicated with digesta diluted 1:3 in DMEM with or without FBS as indicated. **a** 2 or 4 h incubations with digesta (0.1 wt% Fe_2_O_3_ in initial food emulsion) diluted with DMEM +10% FBS **b** 2 or 4 h incubations with digesta (0.1 wt% Fe_2_O_3_ in initial food emulsion) diluted with DMEM without FBS. **c** 4 h incubations with digesta from emulsions containing 0.05 or 0.1 wt% Fe_2_O_3_, diluted 1:3 with DMEM without FBS

The results of a pilot biokinetic study performed as described in detail in the methods section are shown in Fig. 6. Small intestinal digesta from a Fe_2_O_3_ iENM-enabled model food emulsion were diluted in DMEM and applied to the in vitro transwell triculture model of the small intestinal epithelium. Apical and basolateral fluid, as well as cells plus transwell membranes, were collected after incubation for either 2 or 4 h. TEER measurements performed prior to and at the end of the incubation period were not significantly different (data not shown), suggesting that the triculture monolayer remained intact. The resulting samples were then analyzed by ICP-MS for Fe in order to determine the percentage of total Fe_2_O_3_ applied to the apical surface of the cells in the diluted digesta that was either associated with or taken up by cells, and the percent of total Fe_2_O_3_ that passes through or between cells into the basolateral compartment. The results shown in Fig. 6a and b indicate cell uptake and transcytosis after 2 and 4 h from digestae diluted in media with or without FBS. As would be expected, uptake and transcytosis are both greater after 4 h than after 2 h. More importantly, these results suggest that the presence of FBS altered both uptake and transcytosis, which further supports the hypothesis tha the corona formation on the particles will affect their bioactivity. The average percentage (*N* = 2) of total applied Fe_2_O_3_ associated with cells and the membrane when FBS was included in the diluting media was 22.4% at 2 h and 45.7% at 4 h. By comparison, in the absence of FBS, which is the preferred protocol, the mean percentage of cell-assocated Fe_2_O_3_ at 2 and 4 h was 13.0% and 31.1%, respectively. The mean percentage of Fe_2_O_3_ reaching the basolateral compartment when digestae were diluted in media with FBS was 0.18% and 0.77% at 2 and 4 h, respectively. By contrast, mean transcytosis of Fe_2_O_3_ in the absence of FBS was 0.55% and 1.77% at 2 and 4 h.

Finally, Fig. 6c shows results of the uptake of Fe_2_O_3_ by the cells after 4 h incubation with diluted (DMEM only) digestae collected after the exposure of the model food emulsion to the simulated GIT, for systems containing two different initial concentrations of Fe_2_O_3_ ENM (0.05 and 0.1 wt%). Interestingly, whereas the mean percentage of applied Fe_2_O_3_ taken up by cells was subatantially greater at the higher initial ENM concentration (37 vs 22%), the opposite trend was seen in terms of Fe_2_O_3_ transported to the basolateral compartment (0.7 vs 1.1%).

## Discussion

The exposure of consumers to ENMs through consumption of commercial foods and beverages is inevitable, and so it is essential that we have the means to efficiently evaluate their potential biological effects. Here we have presented a prototype integrated platform for such studies, including incorporation of iENMs into model food matrices, simulated GIT exposure, and utilization of a cell culture model that emulates the small intestinal epithelium. We also presented results from utilization of this methodological platform with a case study using a Fe_2_O_3_ ENM and a model food emulsion. It should be noted that the purpose of this study is to present an integrated approach that can be used to study the potential effects of iENMs in vitro, taking into consideration iENM-food-GIT interactions, and not to provide a detailed toxicological and mechanistic investigation for the Fe_2_O_3_ iENMs employed in the case study. A wide variety of additional endpoints beyond the simple live/dead stain and LDH assays presented here would be necessary to fully explore the potential effects of this and any other iENMs. Likewise, positive and negative control particles should, when possible, be employed in such detailed mechanistic studies.

For in vitro studies of iENM toxicity and biokinetics in the GIT, simply dispersing iENMs in culture media is not appropriate, since the physicochemical transformations of iENMs in culture media cannot approximate the complex transformations that would occur during incorporation into the food matrix and subsequent exposure to the physical and chemical environment of the GIT. It was clearly demonstrated in this study that such transformations dramatically influence their biological properties and should not be ignored. Thus, key components of any cellular platform for studying iENM biointeractions must take into consideration food matrix effects and include incorporation of the iENMs into model foods, and subsequently to simulate GIT exposure of the iENM-containing model food. The simple model food employed in the current studies, a protein-stabilized oil-in-water emulsion, is only one of a practically unlimited number of possible food models that might be used. We chose this model for these studies because it is relatively simple, and can easily be modified for future studies to investigate the effects of changes in food composition on iENM toxicity and biokinetics. Moreover, many commercial food and beverage products exist fully or partly as oil-in-water emulsions, including creams, desserts, dips, dressings, sauces, soft drinks, and yogurts. Alternatively, more complete food models, such as whole milk or nutritional drinks such as EnsurePlus®, can be employed to better represent the typical fed state. Even more complex or whole foods could also be used, such as breads, cereals, cookies, fruits, meats, potatoes, rice, or vegetables. Developing more realistic standardized food models to assess potential matrix effects based on various types of diets is of great interest and should be pursued as part of future research in this area.

Another variable factor that could affect the transformation of iENMs in food and during digestion (and thus their bioactivity, toxicity and biokinetics) is the method by which the iENMs are incorporated into the food model. In the work presented here, we chose to first create a monodisperse suspension in DI water and then incorporate that into our liquid food model by simply vortexing. However, a number of alternative methods are possible. For example, the dry powder ENM might be simply added directly to the food and the combination either stirred or vortexed to mix. The most appropriate method might be that which is actually used by food manufacturers to incorporate iENMs into their products. However, these methods may not be easy to ascertain. Moreover, since a given iENM may be used in a number of different foods in terms of consistency, composition, and preparation method. Thus, standardization of iENM-food incorporation methods could prove difficult.

The in vitro GIT simluator presented here is an approximation of the in vivo process, both physically and chemically. It was designed for investigating simple model foods, and does not include a number of chemicals and enzymes that would normally be present in the human GIT. Moreover, the residence times and pH-time profiles used in the GIT simulation do not accurately mimic physiological conditions. Although some enzymes and chemicals may not be needed for digestion in a simple oil-in-water emulsion food model, they would normally be present regardless of the food model contents, would themselves be processed by digestion, and their breakdown products would contribute to the corona and interfacial properties of the iENMs. Adjustments to GIT simulators operational parameters and conditions beyond the described protocols and chemicals can be done to further enhance physiological relevance.

Furthermore, it should be mentioned that the in vitro triculture model proposed here is a hybrid approximation of the small intestinal epithelium. In fact, no part of the small intestinal epithelium contains endocytes (differentiated Caco-2), goblet cells (HT29-MTX) and M-cells (differentiated caco-2 cells transformed by factors from Raji B cells) together. Peyer’s patches and lymphoid associated endothelium contain enterocytes and M-cells, but no goblet cells, and the function of these areas is primarily immune surveillance, whereas the remaining epithelium is the primary absorptive tissue and consists of goblet cells and enterocytes only, with no M-cells. However, using a hybrid such as the triculture model, where M-cells are relatively rare and scattered (<1%) ensures that most of the model cell system is absorptive epithelium-like, while some M-cells are present to provide some degree of the indiscriminate translocation of luminal contents to the basolateral compartment. Other cellular models can be used as part of the proposed integrated methodology.

Finally, because of the complex composition of all but the simplest food models, detailed characterization of the iENM transformations across the iENM-GIT continuum involves many technical challenges Nevertheless, DLS and laser diffraction analysis combined with TEM, SEM and other imaging modalities as well as emerging chemical analysis techniques such as sp.-ICPMS can help us understand the properties of these complex mixtures and of the iENMs incorporated within them [77, 78]. Omics and corona characterization approaches can also be explored to provide additional information that will facilitate the assessment of nano-biointeractions and to develop structure activity relationships (SAR) [79–81].

## Conclusions

The integrated methodology presented here takes into consideration the interactions of ENMs with the food matrix and their transformations as they pass through the different regions of the GIT, and therefore provides a relatively simple but powerful platform for the in vitro study of the biokinetics and toxicology of iENMs. The development and utilization of standardized model foods and simulated GIT digestion to replicate the transformations that would normally occur in real exposures to iENMs is essential. Likewise, the use of advanced physiologically relevant cell culture models, such as the triculture cell model employed in these studies, that approximate the structure of the real intestinal epithelium will allow us to obtain more meaningful data from toxicity and biokinetics experiments that are essential for assessing the hazards or bioactivities of iENMs.

We have demonstrated the application of this platform in a case study using an example iENM (nano Fe_2_O_3_) and a relatively simple model food (oil-in-water emulsion). This platform should be easily adapted for other iENMs and other food models, or used to study the effects of model food preparation and composition and method used to incorporate iENMs. In addition to simple toxicity and biokinetics studies of iENMs, the platform could also be adapted to study the fate and transport of nano-nutraceuticals, or to evaluate the effects of iENMs on the biokinetics and bioavailability of co-ingested nutrients or drugs.

## Additional file

Additional file 1: Methods: Module 1: Assessment of iENM- food interactions (food matrix effect) using food models (Preparation of ENM dispersions in water, Synthesis of food model emulsion, Preparation of Fe2O3 ENM nano-enabled food model); Module 2: Development and assessment of a three stage GIT simulator including mouth, stomach and small intestinal phases for the digestion of nano-enabled food models (Simulated digestion protocol); Module 3: Development and morphological characterization of an in vitro intestinal epithelial model suitable for iENM biokinetics and toxicity studies (Triculture model cell culture method, Measurement of transepithelial electrical resistance (TEER), Triculture immunostaining and imaging for morphological characterization, Triculture cell model TEM characterization; Cellular biokinetics and toxicity experiments (Biokinetics protocol, ICP-MS analysis of biokinetics samples, Cellular Toxicity studies); Colloidal characterization of ENM dispersions, food model and digestae throughout the GIT (Size distribution characterization, Morphological characterization, Dissolution studies for iENM case study); Pristine ENM synthesis and characterization (Synthesis of Fe2O3 ENMs, Pristine ENM characterization) **Figure S1:** Schematic of biokinetics and toxicity experimental system; **Figure S2:** Schematic of flame spray pyrolysis setup; **Figure S3:** TEM image pristine Fe2O3 ENM; **Figure S4:** Emulsion and digestae morphology by fluorescence microscopy; **Figure S5:** Cytotoxicity of triculture model; **Figure S6:** Dissolution of Fe2O3 along GIT; **Table S1:** Chemicals for simulated digestion; **Table S2:** Simulated digestion stock and working solutions. (DOCX 5948 kb)

## Abbreviations

DLS: Dynamic light scattering
ENM: Engineered nanomaterial
FBS: Fetal bovine serum
GIT: Gastrointestinal tract
GRAS: Generally regarded as safe
ICP-MS: Inductively coupled plasma mass spectroscopy
iENM: Ingested engineered nanomateral
SAR: Structure activity relationship
SEM: Scanning electron microscopy
sp.-ICPMS: Single particle inductively coupled plasma mass spectroscopy
TEM: Transmission electron microscopy
