# Supplementary material for: An integrated methodology for assessing the impact of food matrix and gastrointestinal effects on the biokinetics and cellular toxicity of ingested engineered nanomaterials
Source: Part Fibre Toxicol. 2017 Oct 13;14:40. doi: 10.1186/s12989-017-0221-5 (PMC5640936; doi:10.1186/s12989-017-0221-5)
Supplement: Additional file 1: Methods: — Module 1: Assessment of iENM- food interactions (food matrix effect) using food models (Preparation of ENM dispersions in water, Synthesis of food model emulsion, Preparation of Fe2O3 ENM nano-enabled food model); Module 2: Development and assessment of a three stage GIT simulator including mouth, stomach and small intestinal phases for the digestion of nano-enabled food models (Simulated digestion protocol); Module 3: Development and morphological characterization of an in vitro intestinal epithelial model suitable for iENM biokinetics and toxicity studies (Triculture model cell culture method, Measurement of transepithelial electrical resistance (TEER), Triculture immunostaining and imaging for morphological characterization, Triculture cell model TEM characterization; Cellular biokinetics and toxicity experiments (Biokinetics protocol, ICP-MS analysis of biokinetics samples, Cellular Toxicity studies); Colloidal characterization of ENM dispersions, food model and digestae throughout the GIT (Size distribution characterization, Morphological characterization, Dissolution studies for iENM case study); Pristine ENM synthesis and characterization (Synthesis of Fe2O3 ENMs, Pristine ENM characterization) Figure S1: Schematic of biokinetics and toxicity experimental system; Figure S2: Schematic of flame spray pyrolysis setup; Figure S3: TEM image pristine Fe2O3 ENM; Figure S4: Emulsion and digestae morphology by fluorescence microscopy; Figure S5: Cytotoxicity of triculture model; Figure S6: Dissolution of Fe2O3 along GIT; Table S1: Chemicals for simulated digestion; Table S2: Simulated digestion stock and working solutions. (DOCX 5948 kb) [file 12989_2017_221_MOESM1_ESM.docx]

**Additional file 1**

**Supplementary Methods:**

**Module 1: Assessment of iENM- food interactions (food matrix effect) using food models**

***Preparation of ENM dispersions in water***

Iron oxide ENM dispersions were prepared using a protocol previously described by the authors[1]. Briefly, sonication was performed in deionized water (DI H_2_O) using the critical dispersion sonication energy (*DSE*_cr_), which has been determined as previously described for each ENM [1,2]. ENMs were dispersed at 2.0 mg cm^-3^ in 5 ml of DI H_2_O in 15 ml conical polyethylene tubes, by sonication using a calorimetrically calibrated cup horn Branson Sonifier S450-A (Branson Ultrasonics Corporation, Danbury, CT) (maximum power output 400 W at 60 Hz, continuous mode, output level 3, power delivered to sample: 1.25 W).

***Synthesis of food model emulsion***

A stock oil-in-water emulsion consisting of 10 wt% corn oil and 90 wt% aqueous phase (1.0 wt% whey protein emulsifier in 5 mM pH 7.0 phosphate buffer) was created by blending for 2 min using a high-speed blender (M133/1281-0, Biospec Products, Inc.). The resulting coarse emulsion was then passed three times through a high pressure homogenizer (M110Y, Microfluidics, Inc) with a 75 µm interaction chamber (F20Y) at 11,000 psi as described previously [3,4].

***Preparation of Fe_2_O_3_ ENM nano-enabled food model***

Initial stock ENM dispersions in DI H_2_O (or an equal volume of DI H_2_O alone for controls without ENM) were combined with stock 10 wt% oil-in-water emulsions and concentrated phosphate buffer solution to achieve a final desired wt% of ENM in a 2.0 wt% corn oil-in-water emulsion with a final phosphate buffer concentration of 5 mM. The volume of ENM dispersion (or DI H_2_O for controls) per ml of final nano-enabled emulsion, $V_{w,ENM}$, was calculated as

$V_{w,ENM}= \frac{\rho\times wf}{C_{w}(1-wf)}$

where *ρ* is the density of the final desired 2.0 wt% emulsion without ENM (measured as 0.9985 g/ml), *wf* is the desired weight fraction of ENM in the final nano-enabled emulsion, and $C_{w}$ is the concentration (mg/ml) of the ENM stock dispersion in DI H_2_O. The volume of concentrated phosphate buffer per ml of final nano-enabled emulsion, $V_{B}$, was then calculated as

$V_{B}= 1.0-\left( V_{w,ENM}+0.2 \right)$

where 0.2 is the volume in ml of 10 wt% oil emulsion needed per ml of final 2 wt% oil. The concentration of the phosphate buffer needed to achieve a final phosphate buffer concentration of 5 mM was calculated as

$C_{B}= 5.0\times{0.8}/{V_{B}}$ mM

To prepare a final volume $V_{f}$ of nano-enabled emulsion, $V_{f}\times V_{w,ENM}$ ml of ENM dispersion in DI H2O was combined with $V_{f}\times V_{B}$ ml of $C_{B}$ mM phosphate buffer and $V_{f}\times0.2$ ml of 10 wt% corn oil-in-water emulsion in a 50 ml conical tube, and the mixture was vortexted at maximum speed for 30 seconds.

**Module 2: Development and assessment of a three stage GIT simulator including mouth, stomach and small intestinal phases for the digestion of nano-enabled food models**

***Simulated digestion protocol***

Chemicals used in the various phases of the GIT simulation process, including suppliers and catalog numbers, are listed in **Supplementary Table 1**. Chemical components of all stock and working solutions, and final molarity and osmolarity are given in **Supplementary Table 2.** All aqueous solutions were prepared with sterile molecular grade deionized water (Corning). Phosphate buffer (5 mM, pH 7.0), artificial saliva stock solution (ASSS), simulated gastric fluid stock solution (SGFSS) and Simulated intestinal fluid stock salt solution (SIFSSS) were prepared in advance and stored for up to 1 month at 4 °C. To prepare the phosphate buffer 0.6 g of potassium phosphate was dissolved in 150 ml of water, pH was adjusted to 7.0, and additional water was added to bring the volume to 1L. ASSS was prepared by stirring the ingredients listed in Table 4 with 100 ml water, sterile filtering (0.2 µm bottle filter, Thermo Scientific), and diluting to 1L with additional sterile water. SIFSSS was prepared by stirring the ingredients listed in Table 4 with 150 ml sterile water, and sterile filtering the resulting solution.

The Artificial Saliva Working Solution (ASWS) and Simulated Intestinal Fluid Bile Salt Solution (SIFBSS) were prepared on the day before the digestion. ASWS was prepared by combining 0.6g porcine gastric mucin type II with 20 ml ASSS, and stirring overnight at room temperature. SIFBSS was prepared by combining 0.1875g of porcine bile extract with 3.5 ml of 5 mM phosphate buffer at pH 7.0 and stirring overnight at room temperature.

On the day of the digestion, forty minutes before the estimated start of the Stomach phase, the Simulated Gastric Fluid Working Solution (SGFWS) was prepared by stirring 0.064g of Porcine pepsin in 20 ml of SGFSS. The Mouth phase was then initiated by combining in a glass Erlenmeyer tube 20 ml of nano-enabled or control food emulsion with 20 ml of ASWS, both previously warmed to 37°C. The mixture was titrated to pH 6.8 by adding the necessary volume of either sodium hydroxide or hydrochloric acid solutions, and then placed in an incubator shaker (Incubating Orbital Shaker, VWR) for two minutes, at 100 rpm at 37°C.

The Gastric phase followed, with the mixing of 20 ml of the Mouth phase product to 20 ml of SGFWS, previously warmed to 37°C. The mixture was also placed in a glass Erlenmeyer, in a shaker incubator at 100 rpm and at 37°C, and incubated for two hours.

Prior to the start of the Intestinal phase, the Simulated Intestinal Fluid Lipase Solution (SIFLS) was prepared by stirring 0.06g into 2.5ml of 5 mM phosphate buffer for thirty minutes and then warmed to 37°C. After the end of the Stomach incubation period, 30 ml of Stomach phase product were combined to 51.9 ml of 5 mM phosphate buffer at 37°C in a glass beaker and placed in a water bath, also at 37°C. For the Intestinal phase, a pH Stat (TitroLine 7000, SI Analytics) measurement probe, titration probe and an overhead stirrer (Petite Digital Stirrer, Caframo) were placed in contact with the solution in the beaker. The sample was then manually titrated to pH between 6.995 and 6.999 before the addition of each one of the solutions involved in the Intestinal phase, which are, in this order, 1.5 ml of SIFSSS, 3.5 ml of SIFBSS and 2.5 ml of SIFLS, all previously warmed to 37°C. The pH Stat was then initiated for a titration period of two hours, maintaining the pH constant at 7.00 with the addition of the necessary volume of a 0.25 N aqueous solution of sodium hydroxide.

100 IU/ml Penicillin and 100 ug/ml Streptomycin were added to the final digesta samples. Samples were stored at 4°C in 50 ml Falcon tubes for biokinetics and toxicity studies on the following day.

**Module 3: Development and morphological characterization of an *in vitro* intestinal epithelial model suitable for iENM biokinetics and toxicity studies**

***Triculture model cell culture method***

Cryopreserved samples of Caco-2, HT29-MTX and Raji B cell lines were obtained from Sigma-Aldrich. Frozen samples were thawed by immersion in a 37 °C water bath, diluted in 10 ml of the appropriate growth media and pelleted at 100 × *g*. Pellets were re-suspended in 10 ml of growth media and transferred into 75 cm^2^ cell culture flasks with vented caps (Falcon-Becton Dickinson). Caco-2 and HT29-MTX cells were grown in high-glucose DMEM supplemented with 10% heat-inactivated fetal bovine serum (FBS), 10 mM HEPES buffer, 100 IU/ml Penicillin, 100 µg/ml Streptomycin and non-essential amino acids (1/100 dilution of 100 X solution, ThermoFisher). Raji B cells were cultured in RPMI 1640 media supplemented with 10% FBS, 10 mM HEPES buffer, 100 IU/ml Pennicillin and 100 µg/ml Streptomycin. The adherent cell lines Caco-2 and HT29 were subcultured when cells reached 70% confluence, and the non-adherent/suspension Raji B cells were subcultured when cell density reached 1 million cells/cm^3^. To subculture, adherent cells were washed once with TrypLE™ Express (ThermoFisher) then incubated with 5 ml of fresh TrypLE at 37 °C until cells detached from the bottom of the flask (7-8 min). 10 ml of complete DMEM media was then added to the flasks and mixed with the detatched cells and TrypLE™. The re-suspended cells were then centrifuged at 100 × *g* to pellet the cells, and the pellets then resuspended in fresh complete DMEM media at a concentration of 3 × 10^5^ cells/cm^3^. Fresh 75 cm^2^ flasks were then seeded with 1 ml of the cell suspension and 10 ml fresh complete media. Raji B cells were subcultured by pelleting cells at 100 × g, resuspending pellets in complete RPMI media at a concentration of 1 × 10^6^ cells/cm^3^, and inoculating fresh 75 cm2 flasks with 1 ml of the suspension and 10 ml of fresh complete RPMI media.

To prepare triculture transwells, Caco-2 and HT-29 cells resuspended as described above (at 3 × 10^5^ cells/cm^3^) were combined in a ratio of 3:1 (Caco-2:HT29-MTX) 1.5 ml of the cell mixture was seeded in the apical chamber, and 2.5 ml of complete DMEM media was added to the basolateral compartment of a 6 well transwell plate (specify product, manufacturer). The first change of media for the cells in transwells was done four days after seeding, and repeated every other day until the plates were 10 days old. From culture days 10 to 15, the media was changed everyday. When the cells had reached fifteen days of age, the media in the basolateral compartment was substituted for 2.5ml of a suspension of Raji B cells at a concentration of 1 million cells/ml in 1:1 DMEM:RPMI complete media. This procedure was repeated for a second day of Raji B cell treatment, completing a total of seventeen days of culture for the transwell plates, when they were ready to be used for toxicity or biokinetics experiments.

***Measurement of transepithelial electrical resistance (TEER)***

Transwell plates were allowed to equilibrate to room temperature prior to measuring TEER. TEER was measured using an EVOM2 Epithelial Volt/Ohm Meter with a Chopstick Electrode Set (World Precision Instruments). Electrodes were sterilized by rinsing in 70% ethanol and allowed to air dry prior to each use. For each transwell insert measurements were made at three different insert locations and averaged. Measurements were corrected by subtracting resistance measured in a blank transwell (with media in basolateral and apical compartments, but no cells), and reported as the product of measured resistance and transwell insert membrane area (i.e., Ω·cm^2^)

***Triculture immunostaining and imaging for morphological characterization***

Triculture cells were examined by immunofluorescence staining for Mucin 2 (MUC2), the major intestinal mucin, and markers found on M-cells, including Glycoprotein 2 (GP2), Sialyl Lewis A antigen (a.k.a. Ca19-9), and Galectin 9. Unconjugated primary antibodies used included mouse monocolonal anti-MUC2 (Abcam, ab11197), rabbit polyclonal anti-GP2 (NOVUS, NBP1-86070), goat polyclonal anti-Galectin 9 (Santa Cruz, sc-19294), and mouse monoclonal anti-Sialyl Lewis A antigen (Abcamm ab15146). Secondary antibodies used for labeling anti-MUC2, anti-GP2, anti-Galectin 9 and anti-Sialyl Lews A antigen primaries were goat anti-mouse IgG AlexaFlour® 488 (ThermoFisher, A-11001), goat anti-rabbit IgG AlexaFlour® 594 (ThermoFisher, A-11012), chicken anti-goat IgG AlexaFluor® 594 (ThermoFisher, A-21468), and chicken anti-mouse IgG AlexaFlour® 488 (ThermoFisher, A-21200), respectively.

Transwell inserts were washed 2 x 2 ml PBS and transferred to a fresh 6-well plate with 4% formaldehyde (2 ml basolateral, 1 ml apical compartment) and incubated at room temperature for 15 minutes. Inserts were transferred to another fresh plate washed 5 times for 2 min with PBS (2 ml basolateral, 1 ml apical compartment). PBS was replaced with 0.2 % Triton-X 100 and cells were incubated for 20 minutes at room temperature to permeabilize the cell membranes. Membranes with cells were then removed from the inserts using a scalpel, and placed in a fresh 6-well plate with 1.5 ml blocking buffer (PBS + 1% BSA and either 10% chicken serum for combined Galectin 9 and Sialyl Lews A antigen staining, or 10% goat serum for combined MUC2 and GP2 staining). All blocking buffer and antibody solutions were filtered with an 0.2 μm syringe filter immediately prior to use. Inserts were incubated in blocking buffer at 4^o^C for 1 hour with gentle rocking. Blocking solution was replaced with 1.5 ml of the primary antibody pair (Galectin 9 + Sialyl Lewis A antigen, or MUC2 + GP2) in the corresponding blocking buffer (all primary antibodies were at 5 μg/ml), and incubated overnight at 4 ^o^C with gentle rocking. Membranes were then washed 5 times for 10 min each with 2 ml PBS and gentle rocking. After the last wash PBS was replaced with 1.5 ml of the corresponding secondary antibody combination (all secondary antibodies were applied at 10 μg/ml) in the corresponding blocking buffer plus 10 μg/ml Hoechst 33342 nuclear counterstain (ThermoFisher, H3570), and inserts were incubated with gentle rocking at 4 ^o^C overnight. Inserts were then washed 5 times for 10 min each with 2 ml PBS and gentle rocking. Membranes were placed on a glass slide and a cover slip placed over the cells. Confocal fluorescence images were acquired using a BD Pathway 855 Bioimaging System.

***Triculture cell model TEM characterization***

Transwell membranes were washed twice with PBS, cut from inserts with a scalpel and immersed in a routine fixative of 2.5% Glutaraldehyde, 1.25% formaldehyde and 0.03% picric acid in 0.1 M sodium cacodylate buffer (pH 7.4). After overnight fixation at room temperature, membranes were washed in 0.1M cacodylate buffer and postfixed with 1% Osmiumtetroxide (OsO4)/1.5% Potassiumferrocyanide(KFeCN6) for 1 hour, washed 2x in water, 1x in maleate buffer (MB), and 1x and incubated in 1% uranyl acetate in MB for 1hr followed by 2 washes in water and subsequent dehydration in grades of alcohol (10min each; 50%, 70%, 90%, 2x10min 100%). The samples were then put in propyleneoxide for 1 hr and infiltrated overnight in a 1:1 mixture of propyleneoxide and TAAB Epon (Marivac Canada Inc. St. Laurent, Canada). The following day the samples were embedded in TAAB Epon and polymerized at 60 ^o^C for 48 hrs.

Ultrathin sections (about 60nm) were cut on a Reichert Ultracut-S microtome, picked up on to copper grids stained with lead citrate and examined in a JEOL 1200EX Transmission electron microscope or a TecnaiG² Spirit BioTWIN and images were recorded with an AMT 2k CCD camera.

**Cellular biokinetics and toxicity experiments**

***Biokinetics protocol***

Biokinetics studies were performed using the final small intestinal digesta resulted from the GIT simulator (module 2) in serum-free DMEM to the apical compartment of the transwell triculture, and incubating for the desired time period. Following incubation, fluid and washings from the apical and basolateral compartments, and the cells on the transwell membrane, were collected for analysis by ICP-MS.

The resulted from GIT simulator small intestinal digesta was diluted 1:3 in DMEM (without FBS). Apical media was aspirated from triculture transwell inserts, cells washed 2 x 1 ml of PBS, inserts transferred to a fresh 6-well plate with 2.5 ml DMEM (without FBS) in the basolateral compartment, and 1.5 ml of the diluted digesta was applied to the apical compartment of each transwell. After incubation at 37 ^o^C for the designated time (2 or 4 h), apical fluid was aspirated and collected. Cells were washed 3 x 2 ml with PBS and washings combined with the apical fluid sample. Basolateral fluid was aspirated and collected, and basolateral compartments were washed 2 x 2 ml with PBS and washings added to the basolateral compartment sample. The transwell membranes with attached cells were cut from the inserts using a scalpel and placed in a 15 ml tube with 2.5 ml PBS. All samples were stored at 4 ^o^C until analysis by ICP-MS.

***ICP-MS analysis of biokinetics samples***

Aqueous samples from the transwell compartments were concentrated to approximately 2.5 mL and digested for 45 min on a hot block digester (SCP Science, Champlain NY) with 500 μL of HNO_3_. The digests were diluted to 6 mL and filtered (45 µm filter Labsciences, Inc) before analysis. The Fe content in each sample was determined by Inductively Coupled Plasma-Mass Spectrometry (ICP-MS) as follows. The samples were analyzed by ICP-MS (Agilent 7500ce, Santa Clara, CA) for Fe content (56 amu) which was quantified against a four-point calibration curve that had been previously evaluated for accuracy and linearity. Analytical blanks, matrix blanks, and continuing calibration verification samples were included in each sequence.

***Cellular Toxicity studies:***

Cellular toxicity of the small intestinal digesta (generated from the nano-enabled emulsion) was assessed in the transwell triculture cell model by several methods.

*LDH Assay* : Lactate Dehydrogenase activity in the supernatants of triculture wells exposed to small intestinal digesta or digesta diluted 1:3 with serum-free cell culture media (DMEM without phenol red) was assessed using the Pierce™ LDH assay kit (ThermoFisher). To measure spontaneous LDH activity, 45 minutes prior to the end of the incubation period, 150 μl of media was removed from the apical compartment of an untreated well (incubated with DMEM only) and replaced with water. To measure the maximum LDH activity, 45 minutes prior to the end of incubation period 150 μl of media was removed from the apical compartment of the untreated well and replaced with 150 μl lysis buffer (9% Triton X-100). At the end of the incubation period to ensure complete lysis, cells in the maximum LDH well were disrupted using a cell scraper. 50 μl of apical compartment fluid for each test exposure, a corresponding control containing the same digesta, digesta dilution or media that had not been applied to cells, and spontaneous and maximum LDH controls were transferred in triplicate to a 96 well plate. 50 μl of reaction mixture (containing substrate), prepared according to the manufacturer’s instructions, was added to each well and mixed by tapping. The plate was then incubated for 30 minutes at room temperature, protected from light. After incubation, 50 μl of Stop solution was added to each well and mixed by tapping. Absorbance at 490 and 680 nm was measured using a SpectraMax M5 plate reader (Molecular Devices). Absorbance was adjusted using the equation:

$$LDH=A_{490}-A_{680}$$

Adjusted absorbance was corrected for media affects using the equation:

$${LDH}^{'}={LDH}_{+cells}-{LDH}_{-cells}$$

Finally, % cytotoxicity was calculated as:

$$\%{Cytotoxicity}_{\text{Tx}}=100\times\frac{{LDH}_{\text{Tx}}^{'}-{LDH}_{\text{Spont}}^{'}}{{LDH}_{\text{Max}}^{'}-{LDH}_{\text{Spont}}^{'}}$$

Live/Dead and ROS fluorescence microscopy: Transwell triculture monolayers were stained and imaged, after incubation with digestae or diluted digesta, for assessment of cell viability by confocal microscopy. Cell viability was assessed by staining with the cell membrane-impermeant dye Ethidium Homodimer-1 and enzymatic activity was assessed by staining with Calcein AM. Transwell inserts were washed once with 0.5 ml of PBS, transferred to fresh plates and incubated for 30 minutes at 37º C with 500 μl of 2 μM Calcein AM (ThermoFisher), 4 μM Ethidium Homodimer-1 (ThermoFisher) and 2 μg/ml Hoechst nuclear dye (ThermoFisher) in PBS. Membranes were washed twice with 0.5 ml of PBS, cut from the inserts and mounted on glass slides. Images were acquired using a scanning cytometer/spinning-disc confocal fluorescence microscope (BD 855 Bioimager)

In a second dual staining method, transwell triculture monolayers were analyzed for Reactive Oxygen Species (ROS) generation and membrane integrity. ROS generation was evaluated by staining with CellROX Orange (ThermoFisher), and cell membrane damage was assessed using the membrane-impermeant dye SYTOX Green (ThermoFisher). Transwells were washed once with 0.5 ml of PBS, transferred to fresh plates and incubated for 30 minutes at 37º C with 500 μl of PBS with 10 uM CellROX Orange, 2.0 μM SYTOX green and 0.5 μg/ml Hoechst 33342 (ThermoFisher) dilution of 100 nM MitoTrackerRed-CMXRos, 10 μM CellROX Green, and Hoechst 2 μg/ml (for nuclear staining and identification). After the incubation period the membranes were washed twice with 0.5 ml of PBS, cut out from the insert and mounted on glass slides with coverslips for immediate imaging on confocal microscope (BD 855 Bioimager).

**Colloidal characterization of ENM dispersions, food model and digestae throughout the GIT**

***Size distribution characterization***

Fe_2_O_3_ water dispersions, the nano-enabled food model (emulsion) and digestae from each phase of the simulated GIT digestion were analyzed by dynamic light scattering (DLS) for determination of hydrodynamic diameters (*d*_H_) and polydispersity indices (PdI) using a Zetasizer Nano-ZS (Malvern Instruments, Ltd.). Particle size distributions for all suspensions and digestae were also determined using a laser diffraction device (Mastersizer 2000, Malvern Instruments, Ltd.). To avoid multiple scattering artifacts samples were diluted in either DI H_2_O (for initial Fe_2_O_3_/DI H_2_O dispersion), 5 mM pH 7.0 phosphate buffer (for initial nano-enabled emulsion, mouth and small intestine digesta samples), or DI H_2_O adjusted to pH 2.5 (for stomach digesta samples). Sizes obtained by laser diffraction are reported as the surface-weighted mean diameter, *d*_32_.

***Morphological characterization***

TEM: 5μl of the sample was adsorbed for 1 minute to a carbon coated grid that had been made hydrophilic by a 30 second exposure to a glow discharge. Excess liquid was removed with a filter paper (Whatman #1) and the samples were stained with 0.75% uranyl formate for 30 seconds. After removing the excess uranyl formate with a filter paper, the grids were examined in a JEOL 1200EX Transmission electron microscope or a TecnaiG² Spirit BioTWIN and images were recorded with an AMT 2k CCD camera.

Fluorescence microscopy: Samples were mixed with Nile red dye at 0.1 μM and incubated for 20 min at 37 ^o^C. 10 μl samples were mounted on slides, covered with a coverslip, and then imaged using a BD 880 Bioimager with standard filters for red fluorescence.

***Dissolution studies for iENM case study***

Food model and digestae samples from various phases of the GIT simulator were centrifuged for 2 h at 13500 RPM (19357 x g). After centrifugation, three different phases were visible within the tube and each phase was analyzed for the Fe content. The top phase was composed of fat or oil droplets from the emulsion or digesta and was located at the surface of the supernatant. The fat/oil droplets were separated from the supernantant by slowly adding liquid nitrogen on top of the solution, freezing only the top layer. The frozen layers were then removed with a plastic spatula, transferred to a digester tube, and then weighed. Blanks were prepared following the same procedure but using Milli-Q water and liquid nitrogen. The remainder of the solutions were centrifuged again for 2 h; the supernatant and pellet were separated and transferred to separate digester tubes and were then weighed. The fat/oil droplets and the pellets were acidified with 2 mL of HNO_3_. The supernatants were concentrated to 2 mL and acidified with 2 mL of HNO_3_. The samples were then digested for 45 min at 115 °C and were then diluted to 25 mL with Milli-Q water. The Fe content in each sample was determined by Inductively Coupled Plasma-Mass Spetrometry (ICP-MS) as follows. The samples were analyzed by ICP-MS (Agilent 7500ce, Santa Clara, CA) for Fe content (56 amu) which was quantified against a four-point calibration curve that had been previously evaluated for accuracy and linearity. Analytical blanks, matrix blanks, and continuing calibration verification samples were included in each sequence. Blanks samples analyzed by ICP contained non-detectable levels of Fe.

**Pristine ENM synthesis and characterization**

***Synthesis of Fe_2_O_3_ ENMs***

The Fe_2_O_3_ nanoparticles were made with the Harvard Versatile Engineer Nanomaterial Generation System (VENGES) [5–7] a Flame Spray Pyrolysis (FSP) based platform. In summary, a precursor solution, which contains dissolved organometallic compounds in a high enthalpy combustible solvent, is fed through a stainless-steel capillary tube at a controlled flow rate. Oxygen flow disperses the liquid precursor solution into fine droplets, which are easily combusted by a small pilot flame. This results in the full conversion of the liquid precursor’s organic constituents into vapor, which as it is cooling down condenses to metal oxide nanoparticles [8]. The nanoparticle properties, are fully controlled by the operational parameters (precursor concentration, precursor feeding rate, dispersion gas) and the results are consistent and reproducible [9]. The nanoparticles are collected on a water-cooled stainless steel mesh, supported on a glass fiber filter (Whatmann, 25.5 cm Ø). If larger sizes are desired a quartz enclosure could be used to maintain the temperature profile that will further grown the ENMs. In that case a sheath gas flow is used to prevent particle aggregation and collision with the enclosure walls. The flame-spray set-up is depicted in **Supplementary Figure 2**.

The Fe_2_O_3_ nanoparticles precursor was prepared with a 0.34 M Fe(III) acetylacetonate (Aldrich, > 97%) in a o-xylene : acetonitrile (Sigma-Aldrich, > 99.5%), 3:1 volume ratio, solvent mixture [10]. The precursor was fed at 12 mL/min with 5 L/min dispersion and 3 L/min sheath oxygen [10].

***Pristine ENM characterization***

Brunauer–Emmett–Teller (BET) analysis

Specific surface area, *SSA*, for Fe_2_O_3_ ENM was determined by the nitrogen adsorption/Brunauer-Emmett-Teller (BET) method using a NovaTouch LX4 (Quantachrome, Boynton Beach, Fl). The particles were degassed under vacuum at 300 C for and a 5 point BET surface adsorption was carried out with nitrogen gas in liquid nitrogen temperature. Equivalent primary particle diameter, *d*_BET_, was calculated, assuming spherical particles, as

$d_{BET}=\frac{6}{SSA\times\rho_{\text{p}}}$,

where *ρ*_p_ is the particle density, which was obtained for each particle from the densities of component materials, at 20°C, reported in the CRC handbook of Chemistry and Physics [11].

X-Ray Diffraction (XRD) analysis

XRD analysis was done with a Bruker D8 VENTURE equipped with a PHOTON-100 CMOS detector, high brilliance Mo/Cu IµS microfocus X-ray sources and Oxford Cryosystream 800 series low temperature device. The Scherer formula was used to estimate the crystalline size (*d*_XRD_).

TEM imaging

A suspension of the ENMs was made and the the drop-casting method was used to adhere the particles on a TEM grid (TedPella, Redding, CA #01822). In brief the suspension was prepared according the protocol as previously described [2], and 10 µl of suspension was applied to the TEM grid for 3 minutes. Filter paper was then used to remove excess water and the grid was left to dry. Imaging was performed with a Jeol 2010.

Endotoxin assay

ENMs were tested for endotoxin levels using the EndoZyme® recombinant factor C (rFD) assay (Hyglos, Germany) according to manufacturer’s instructions. In Brief, 10 ug/ml suspensions of ENMs as well as endotoxin standard dilutions and ENM suspensions spiked with 0.5 EU/ml endotoxin were prepared in endotoxin-free water. Samples, spiked samples and standard dilutions were dispensed into a pre-warmed (37C) 96 well plate (100 ul/well), and mixed with 100 ul assay reagent (8:1:1 ratio of assay buffer, enzyme, and substrate). Fluorescence (Ex 380, Em 440) was measured at t=0 and at 90 minutes. Endotoxin levels were calculated from sample fluorescence using a standard curve equation generated from standard endotoxin dilutions.

**Additional file 1: Figures:**


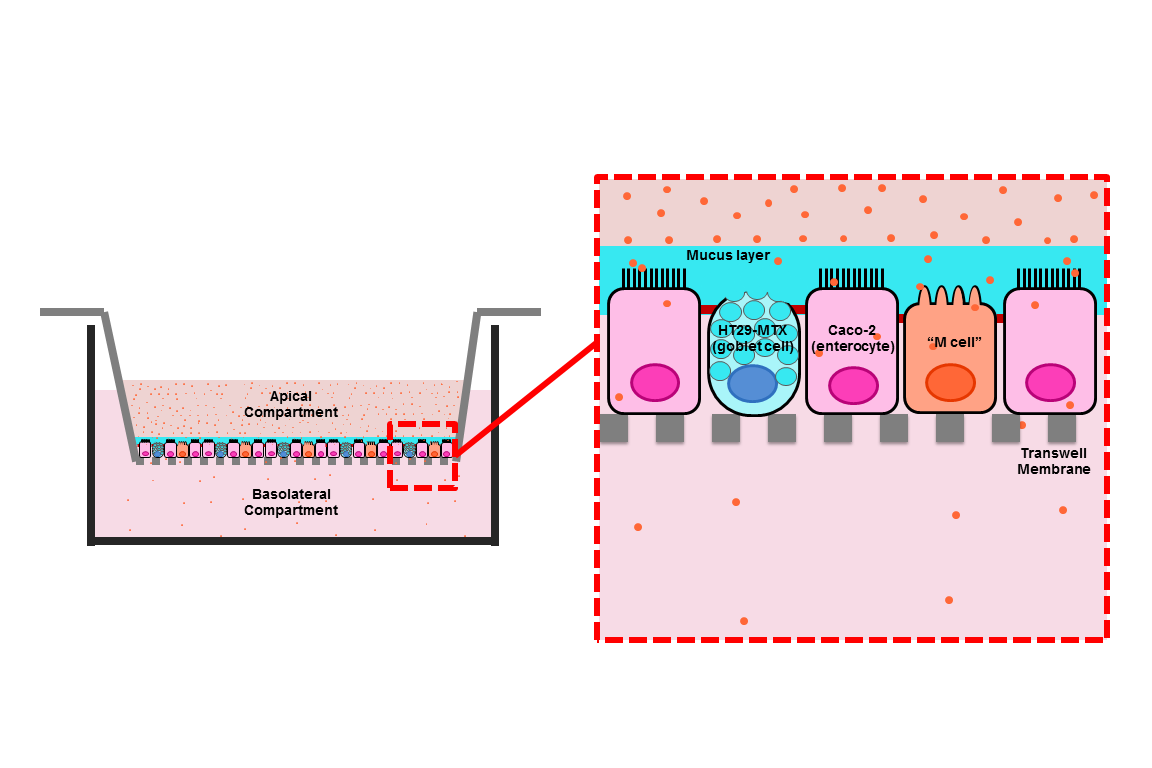


**Figure S1 | Schematic of biokinetics and toxicity experimental system.** Intestinal epithelium triculture model was grown on transwell inserts. Digestae from simulated GIT digestion was applied to the apical surface.


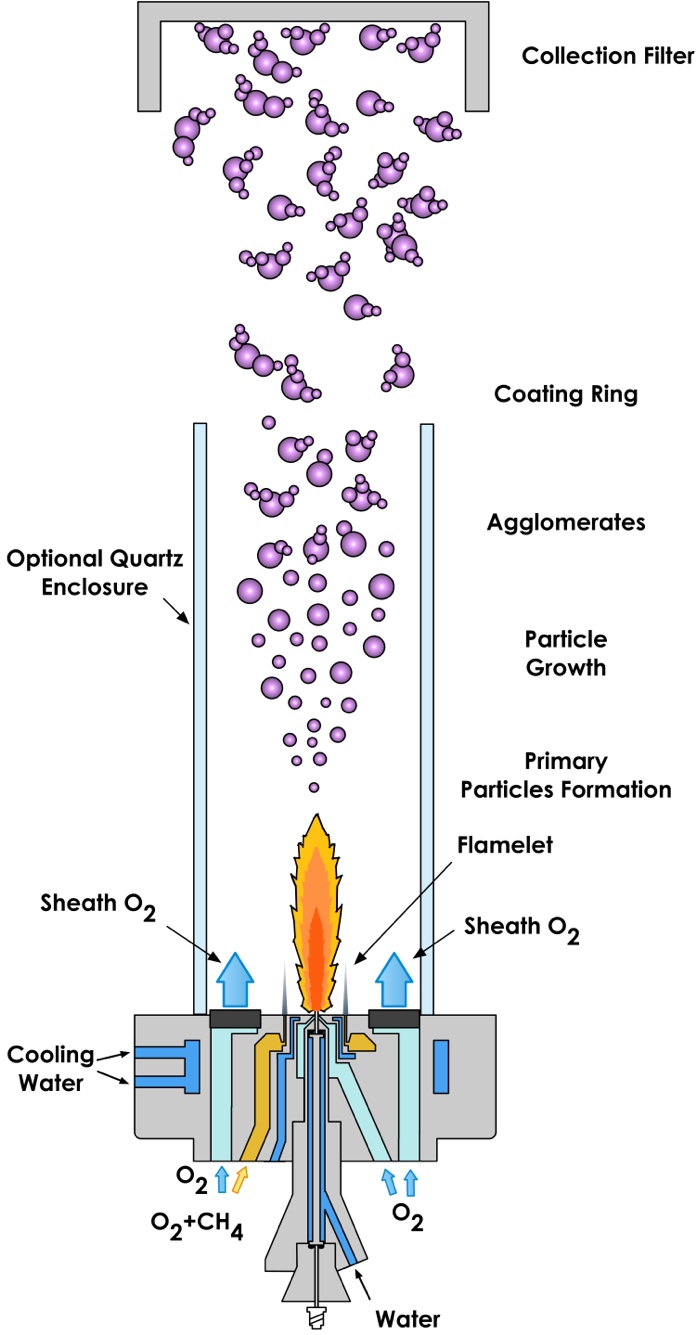


**Figure S2 | Schematic of flame spray pyrolysis setup**

**
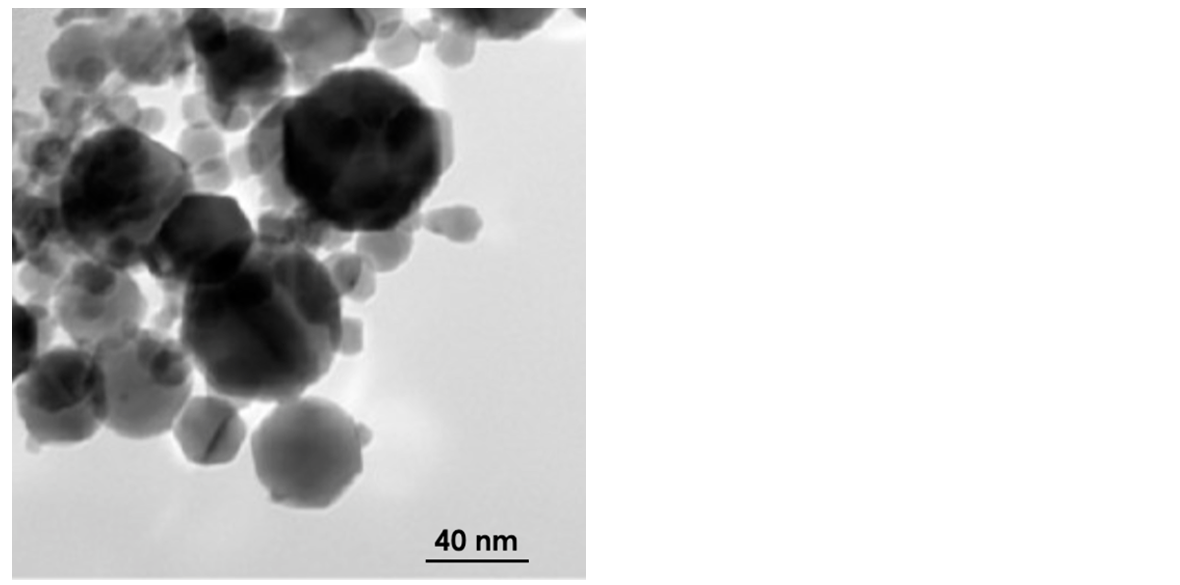
**

**Figure S3 | TEM image pristine Fe_2_O_3_ ENM**


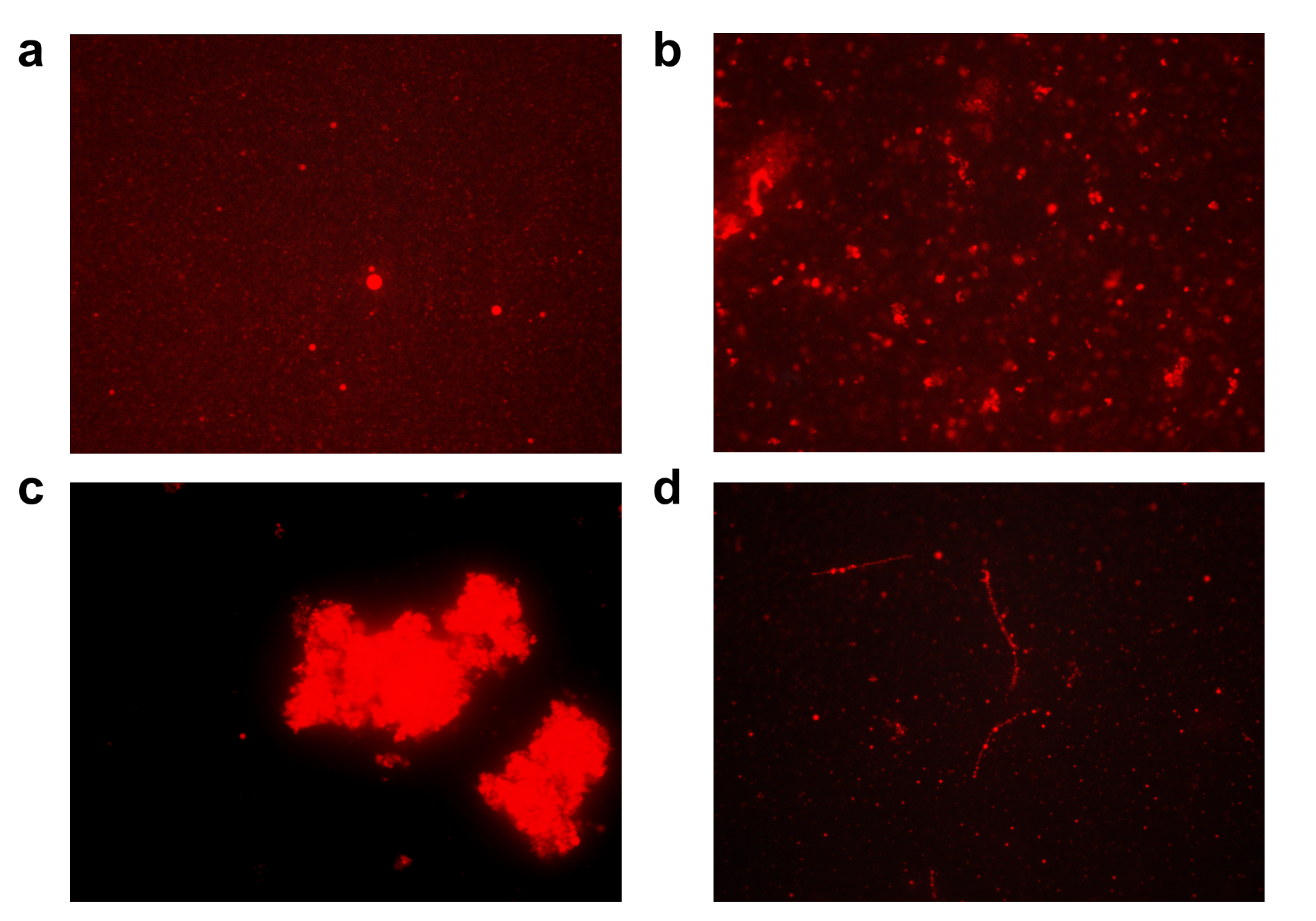


**Figure S4 | Emulsion and digestae morphology by fluorescence microscopy** Samples of emulsion and digestae were stained with Nile Red lipophilic fluorescent stain and imaged by confocal microscopy. **a,** nano-enabled (0.1 wt% Fe_2_O_3_ ENM) food model. **b,** mouth digesta. **c,** Stomach digesta. **d,** small intestinal digesta.


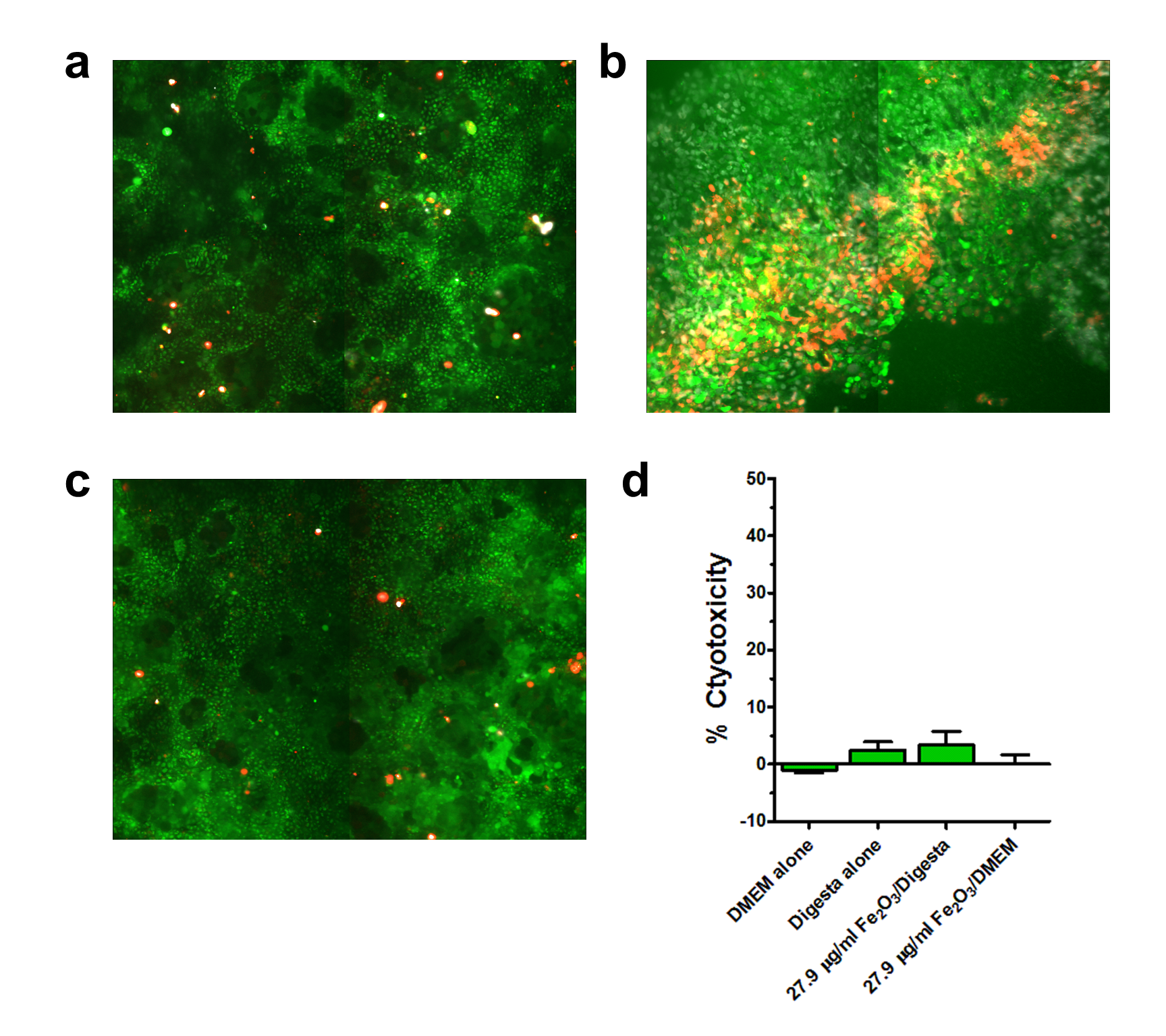


**Figure S5 | Cytotoxicity of triculture model a,** Live/dead staining after 8 hours with DMEM/FBS. **b,** Live/dead staining after 8 hours with undiluted digesta. **c,** Live/dead staining after 8 hours with digesta diluted 1:3 in DMEM without FBS. **d,** LDH cytotoxicity after 8 hours with DMEM alone, diluted digesta of food emulsion without ENM, diluted digesta of food emulsion with final concentration of 27.9 ug/ml Fe_2_O_3_ ENM, and DMEM with 27.9 ug/ml Fe_2_O_3_ ENM .


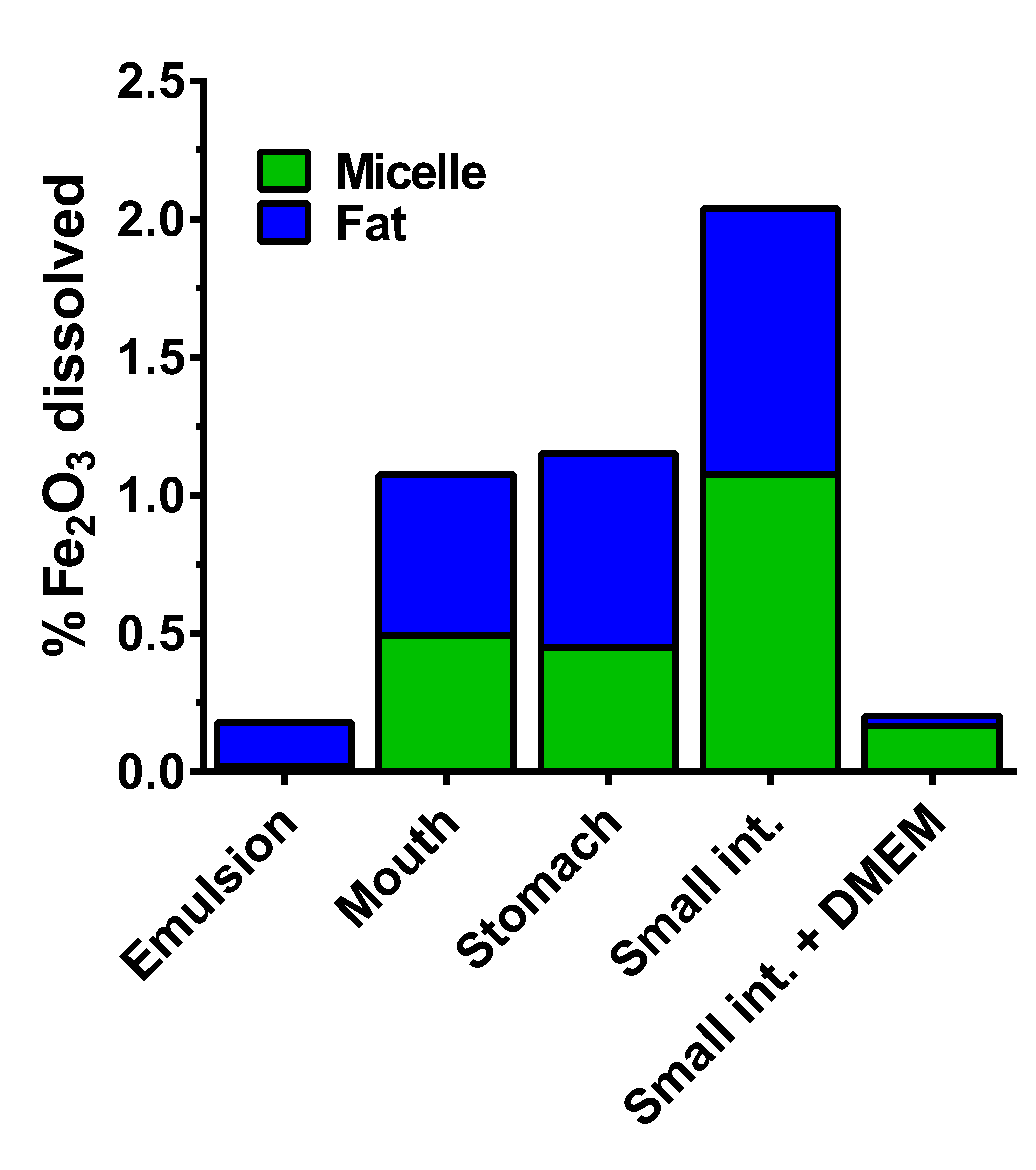


**Figure S6| Dissolution of Fe_2_O_3_ along GIT** Emulsion and digestae were centrifuged and separated into fat, micelle and pellet, and Fe measured in each fraction by ICP-MS

**Additional file 1: Tables:**

**Table S1 Chemicals for simulated digestion**

| **Chemicals** | **Supplier** | **Catalog/part number** |
| --- | --- | --- |
| Sodium chloride | Fisher | S640-3 |
| Ammonium nitrate | Sigma | A9642-500G |
| Potassium phosphate | Sigma | P3786-100G |
| Potassium chloride | Sigma | P9333 |
| Potassium citrate (monohydrate) | Sigma | P1722 |
| Uric acid sodium salt | Sigma | U2875-5G |
| Urea | Sigma | 51456 |
| Lactic acid sodium salt (Sodium DL-lactate) | Sigma | 71720-5G |
| Porcine gastric mucin type II | Sigma | M2378-100G |
| Hydrochloric acid | Fisher | A144-212 |
| Pepsin (porcine) | Sigma | P7000-25G |
| Calcium Chloride (dihydrate) | Sigma | C5080 |
| Lipase (porcine) | Sigma | L3126-100G |
| Bile salts (porcine bile extracts) | Sigma | B8631-100G |

**Table S2 Simulated digestion stock and working solutions**

| **Solution** | **Chemical** | **Mass/Volume** | **MW** | **Molarity** | **N ions** | **Osmolarity**  **(Osm)** |
| --- | --- | --- | --- | --- | --- | --- |
| **5 mM Phosphate buffer pH 7.0** |  |  |  |  |  |  |
|  | Potassium Phosphate | 0.6 g | 136.09 | .005 | 2 | .010 |
|  | titrants (HCl, NaOH) |  |  |  |  | negl. |
|  |  |  |  |  |  |  |
|  |  |  |  |  |  | Total: 0.010 |
| **Artificial Saliva Stock Solution (ASSS)** |  |  |  |  |  |  |
|  | Sodium chloride | 1.594 g | 58.44 | .0273 | 2 | .0546 |
|  | Ammonium nitrate | 0.328 g | 80.05 | .0041 | 2 | .0082 |
|  | Potassium phosphate | 0.636 g | 136.09 | .0047 | 2 | .0094 |
|  | Potassium chloride | 0.202 g | 74.55 | .0027 | 2 | .0054 |
|  | Potassium citrate (monohydrate) | 0.308 g | 324.41 | .0009 | 2 | .0018 |
|  | Uric acid sodium salt | 0.021 g | 190.09 | .0001 | 2 | .0002 |
|  | Urea | 0.198 g | 60.06 | .0033 | 1 | .0066 |
|  | Lactic acid sodium salt (Sodium DL-lactate) | 0.146 g | 112.06 | .0013 | 2 | .0026 |
|  |  |  |  |  |  | Total: 0.0888 |
| **Artificial Saliva Work Solution (ASWS)** |  |  |  |  |  |  |
|  | ASSS | 20 ml |  |  |  | 0.0888 |
|  | Porcine gastric mucin type II | 0.6g |  |  | 1 | negl. |
|  |  |  |  |  |  | Total: 0.0888 |
| **Simulated Gastric Fluid Stock Solution (SGFSS)** |  |  |  |  |  |  |
|  | Sodium chloride | 2 g | 58.44 | 0.342 | 2 | 0.684 |
|  | Hydrochloric acid | 7 ml  ~37.25% | 36.4 | 10.223 | 2 | 0.072 |
|  |  |  |  |  |  | Total: 0.756 |
| **Simulated Gastric Fluid Working Solution (SGFWS)** |  |  |  |  |  |  |
|  | SGFSS | 20 ml |  |  |  | 0.756 |
|  | Pepsin (porcine) | 0.064 g |  |  |  | negl. |
| **Simulated Intestinal Fluid Stock Salt Solution (SIFSSS)** |  |  |  |  |  | Total: 0.756 |
|  | Sodium chloride | 32.87 g | 58.44 | 3.7497 | 2 | 7.500 |
|  | Calcium Chloride (dihydrate) | 5.5 g | 147.01 | 0.249 | 3 | 0.748 |
|  |  |  |  |  |  | Total: 8.248 |
| **Simulated Intestinal Fluid Lipase Solution (SIFLS)** |  |  |  |  |  |  |
|  | Lipase (porcine) | 0.06 g |  |  |  | negl. |
|  | phosphate buffer 5 mM pH 7 | 2.5 ml |  |  |  | 0.010 |
|  |  |  |  |  |  | Total: 0.010 |
| **Simulated Intestinal Fluid Bile Salt Solution (SIFBSS)** |  |  |  |  |  |  |
|  | Bile salts (porcine bile extracts) | 0.1875g |  |  |  |  |
|  | phosphate buffer 5 mM pH 7 | 3.5 ml |  |  |  |  |
|  |  |  |  |  |  | Total: 0.010 |

**REFERENCES**

1. Cohen J, Deloid G, Pyrgiotakis G, Demokritou P. Interactions of engineered nanomaterials in physiological media and implications for in vitro dosimetry. Nanotoxicology. 2013;7: 417–31. Available: http://www.ncbi.nlm.nih.gov/pubmed/22393878

2. DeLoid GM, Cohen JM, Pyrgiotakis G, Demokritou P. Preparation, characterization, and in vitro dosimetry of dispersed, engineered nanomaterials. Nat Protoc. 2017;12: 355–371. doi:10.1038/nprot.2016.172

3. Zhang R, Zhang Z, Zhang H, Decker EA, McClements DJ. Influence of emulsifier type on gastrointestinal fate of oil-in-water emulsions containing anionic dietary fiber (pectin). Food Hydrocoll. 2015;45: 175–185. doi:10.1016/j.foodhyd.2014.11.020

4. Zhang R, Zhang Z, Zhang H, Decker EA, McClements DJ. Influence of lipid type on gastrointestinal fate of oil-in-water emulsions: In vitro digestion study. Food Res Int. 2015;75: 71–78. doi:10.1016/j.foodres.2015.05.014

5. Demokritou P, Büchel R, Molina RM, Deloid GM, Brain JD, Pratsinis SE. Development and characterization of a Versatile Engineered Nanomaterial Generation System (VENGES) suitable for toxicological studies. Inhal Toxicol. 2010;22 Suppl 2: 107–16. doi:10.3109/08958378.2010.499385

6. Demokritou P, Gass S, Pyrgiotakis G, Cohen JM, Goldsmith W, McKinney W, et al. An in vivo and in vitro toxicological characterisation of realistic nanoscale CeO₂ inhalation exposures. Nanotoxicology. 2013;7: 1338–50. doi:10.3109/17435390.2012.739665

7. Sotiriou GA, Diaz E, Long MS, Godleski J, Brain J, Pratsinis SE, et al. A novel platform for pulmonary and cardiovascular toxicological characterization of inhaled engineered nanomaterials. Nanotoxicology. 2012;6: 680–90. doi:10.3109/17435390.2011.604439

8. Pratsinis SE. Aerosol-based technologies in nanoscale manufacturing: from functional materials to devices through core chemical engineering. AIChE J. 2010;56: 3028–3035. doi:10.1002/aic.12478

9. Mädler L, Stark WJ, Pratsinis SE. Flame-made Ceria Nanoparticles. J Mater Res. 2002;17: 1356–1362. doi:10.1557/JMR.2002.0202

10. Li D, Teoh WY, Selomulya C, Woodward RC, Munroe P, Amal R, et al. Insight into microstructural and magnetic properties of flame-made γ-Fe2O3 nanoparticles. J Mater Chem. The Royal Society of Chemistry; 2007;17: 4876. doi:10.1039/b711705a

11. Haynes WM. CRC Handbook of Chemistry and Physics. 92nd ed. Boca Raton, USA: Taylor & Francis; 2011.
